# Global variations and time trends in the prevalence of primary open angle glaucoma (POAG): a systematic review and meta-analysis

**DOI:** 10.1136/bjophthalmol-2015-307223

**Published:** 2015-08-18

**Authors:** Venediktos V Kapetanakis, Michelle P Y Chan, Paul J Foster, Derek G Cook, Christopher G Owen, Alicja R Rudnicka

**Affiliations:** 1 Population Health Research Institute, St George's, University of London, London, UK; 2 Division of Genetics and Epidemiology, UCL Institute of Ophthalmology, London, UK; 3 NIHR Biomedical Research Centre Moorfields Eye Hospital NHS Foundation Trust, London, UK

**Keywords:** Epidemiology, Glaucoma, Intraocular pressure, Public health

## Abstract

Systematic review of published population based surveys to examine the relationship between primary open angle glaucoma (POAG) prevalence and demographic factors. A literature search identified population-based studies with quantitative estimates of POAG prevalence (to October 2014). Multilevel binomial logistic regression of log-odds of POAG was used to examine the effect of age and gender among populations of different geographical and ethnic origins, adjusting for study design factors. Eighty-one studies were included (37 countries, 216 214 participants, 5266 POAG cases). Black populations showed highest POAG prevalence, with 5.2% (95% credible interval (CrI) 3.7%, 7.2%) at 60 years, rising to 12.2% (95% CrI 8.9% to 16.6%) at 80 years. Increase in POAG prevalence per decade of age was greatest among Hispanics (2.31, 95% CrI 2.12, 2.52) and White populations (1.99, 95% CrI 1.86, 2.12), and lowest in East and South Asians (1.48, 95% CrI 1.39, 1.57; 1.56, 95% CrI 1.31, 1.88, respectively). Men were more likely to have POAG than women (1.30, 95% CrI 1.22, 1.41). Older studies had lower POAG prevalence, which was related to the inclusion of intraocular pressure in the glaucoma definition. Studies with visual field data on all participants had a higher POAG prevalence than those with visual field data on a subset. Globally 57.5 million people (95% CI 46.4 to 73.1 million) were affected by POAG in 2015, rising to 65.5 million (95% CrI 52.8, 83.2 million) by 2020. This systematic review provides the most precise estimates of POAG prevalence and shows omitting routine visual field assessment in population surveys may have affected case ascertainment. Our findings will be useful to future studies and healthcare planning.

## Introduction

Glaucoma is a major public health problem, being the leading cause of irreversible visual impairment worldwide.^1^ Primary open angle glaucoma (POAG) is the most common type of glaucoma accounting for three-quarters (74%) of all glaucoma cases.^2^ A recent review estimated the global number of POAG cases in 2013 at 44 million, rising to 53 million by 2020 due to population ageing.^3^ However, uncertainty about the number of people with POAG still remains, as these global estimates are associated with a twofold difference in CIs (ie, estimates ranged from 31 million to 61 million cases in 2013, increasing to 37 million to 73 million in 2020).^3^ Such uncertainty makes it difficult to accurately plan appropriate health services.

Reasons for this uncertainty are numerous and relate to geographical variations in underlying POAG prevalence, as well geographical representation of surveys to ascertain prevalence, and differences in study methods of case ascertainment. Previous population-based surveys have been carried out largely in White populations.^4^ ^5^ A meta-analysis carried out by our group nearly a decade ago showed higher prevalence in Black populations compared with White populations and Asians, but there were little data on other ethnic groups, such as South and East Asians or Hispanics, at that time.^5^ Since then, many large population based studies have been carried out to examine prevalence of POAG in different ethnic groups and geographical locations.^3^ A recent review showed that the greatest increase of POAG cases is estimated to be in lower income countries, particularly Asia, due to more rapid ageing compared with countries of European ancestry. This will have considerable impact on the total number of cases, as Asia has 60% of the world's adult and aged population. However, these estimates did not examine geographical variation in POAG prevalence within Asia, which may be important as any potential error in estimation is magnified when applied to large population numbers.

Moreover, different methods of ascertaining POAG cases will artefactually alter estimates of population prevalence.^5^ Standardisation of POAG definitions by the International Society for Geographical and Epidemiological Ophthalmology (ISGEO) has sought to establish a survey-specific distribution of optic disc parameters to define ‘normality’, allowing studies in less developed countries to contribute to the world literature on glaucoma prevalence.^6^ These definitions avoid historic over-reliance on elevated intraocular pressure (IOP) being a necessary prerequisite for glaucoma diagnosis, and place greater emphasis on visually significant ‘end organ damage’. However, the potential effect of these definitions on case ascertainment is unknown, and has not been formally investigated.

For these reasons, we have updated our review to include data from a larger number of studies. We have adopted a more inclusive approach than another recent review,^3^ to increase the number of cases and participants, in order to establish with greater precision the strength of the relation between POAG prevalence, ethnicity, age and gender. The increase in numbers also allows trends in POAG prevalence through time and the effect of study design on prevalence to be examined with greater certainty, allowing more accurate estimation of the global/regional prevalence and case burden of POAG currently and over the next decade.

## Methods

### Systematic search strategy

The systematic review was carried out by three investigators (MPYC, ARR and VVK) and followed the Meta-analysis of Observational Studies in Epidemiology guidelines for the reporting of systematic review and meta-analysis of observational studies.^7^ The search reviewed all published papers, letters, abstract and review articles published on MEDLINE, EMBASE and Web of Science electronic databases from January 2005 to October 2014. Papers published before 2005 were identified from our earlier review.^5^ A combination of text words for glaucoma (glaucoma/open angle glaucoma/primary open angle glaucoma/primary glaucoma) and epidemiological terms (incident$/prevalen$/population$/survey$) were used, in addition to related subject headings in MEDLINE, EMBASE and Web of Science.

### Inclusion and exclusion criteria

Studies were included if they provided quantitative estimates of POAG prevalence in population-based surveys, where the geographical, random or clustered population sampling method was clearly defined. Studies were only included if optic disc assessment was carried on all participants (ie, not a subset or random sample). Studies reporting audits of hospital eye departments or clinics, or inviting non-specific volunteers were excluded. Studies were not excluded on the basis of clinical definitions of POAG or methods used to diagnose POAG cases; studies using self-reported diagnosis of glaucoma were excluded. Attempts were made to contact authors for further clarification of details where necessary.

### Studies identified and data extraction

In total, 5434 studies were identified and underwent abstract review; 5282 studies were excluded based on the criteria defined above. The QUOROM statement in online supplementary figure S1 shows the article selection process. A list of excluded papers is available from the authors; 81 studies that reported the prevalence of POAG from a defined population-based survey were included in the analysis (including 46 studies identified previously from our earlier review).^5^ Data from these studies were extracted by three reviewers (MPYC, VVK and ARR), with independent extraction in a subset. Disagreements in data extraction were resolved by discussion.

Data were extracted on a number of key indicators of study quality, identified a priori. These included method of POAG diagnosis (whether the definition was based on visual field (VF) results; whether IOP was included in the case definition), and whether these assessments were carried out in all or a subset of participants, in one or both eyes. Where possible, POAG prevalence excluding cases of pseudoexfoliation syndrome was sought. In studies published after 2000, we also recorded whether the study conformed to ISGEO criteria for POAG diagnosis,^6^ and if so, whether VF assessment was carried out on all participants, a subset of high-risk or a proportion of random participants. Data were also extracted on study response rates, habitation type (urban, rural or mixed) and year of survey (midpoint when a study period was reported). Missing data on survey year were imputed for 13 studies by subtracting 4 years from the year that the article was published (based on the median time to publication, in studies with available data). Data were extracted by gender and ethnic/racial group where available. Ethnicity was classified into the groups listed below, broadly following definitions of the United Nations (UN) and WHO:

- White European ancestry (ie, European, Brazilian, American, Australian, New Zealander)
- East Asian (ie, Chinese, Japanese, Mongolian, South Korean)
- South Asian (ie, Indian, Sri Lankan and Bangladeshi)
- South-East Asian (ie, Singaporean, Myanmarese and Thai)
- Black African Caribbean ancestry (ie, Ghanaian, African American, Black Caribbean, Black British)
- Hispanic or Latino (ie, Hispanic or Latino in USA)
- Other or mixed (ie, Eskimo, mixed South African, indigenous Australian, Qatari, Iranian, mixed non-white).

### Statistical analysis

All statistical analyses were carried out using OpenBUGS (V.3.2.2)^8^ and R (V.3.1.1).^9^ We used Bayesian multilevel mixed-effects binomial logistic regression to investigate the associations between the log odds of POAG in either eye and potential modifying factors, including age, gender, ethnicity, year of survey and study design factors such as methods of diagnosis. Our previous review^5^ demonstrated ethnic differences in the prevalence of POAG with age. Therefore, the effect of age was allowed to differ by ethnic group by including an interaction term in the models. Year of survey was included in the models as a categorical variable with four levels (ie, 1960–1979, 1980–1989, 1990–1999, and 2000 or later). The effect of study design was assessed in two ways: first, by including dummy variables indicating whether VF testing was routinely performed on all participants and whether an IOP criteria were used, allowing for an interaction between the two variables; and second, for surveys conducted since 2000 by including a variable indicating whether a study followed ISGEO classification of glaucoma (and if so, whether VF assessment was carried out on all or a subset of participants) or not. The effect of gender was estimated from a separate model using the subset of studies that reported gender-specific prevalence, adjusting for study design, age, ethnicity and an interaction between age and ethnicity. All analyses included a random component for each study population, to take into account the correlation of prevalence estimates within the same study population. A study population was defined as the same ethnicity examined at the same time in the same geographical location.

Modelled age, gender and ethnic specific prevalence estimates were standardised to studies that routinely used VFs on all participants to diagnose POAG, and applied to UN demographic data for 2015, 2020 and 2025.^10^ We selected the dominant ethnic group for the following UN defined regions (1) Black—Africa and the Caribbean, (2) White—Europe, North America, Western Asia, Australia and New Zealand, (3) Hispanic—Central and Southern America, (4) Other/mixed—Melanesia, Micronesia and Polynesia. More detailed ethnic division was possible for Asia where (5) East Asian was used to represent Eastern and Central Asia, (6) South Asian—Southern Asia, and (7) South-East Asian—South-Eastern Asia. Mid age band prevalence estimates were applied to 5-year population data from 40 years to 90 years, to obtain population numbers with POAG, overall and by region, with associated 95% credible intervals (CrIs). In the population with age 90 years or more, prevalence estimates at 92 years were applied. A full description of all statistical models is available as online supplementary statistical appendix.

## Results

**Figure 1:**
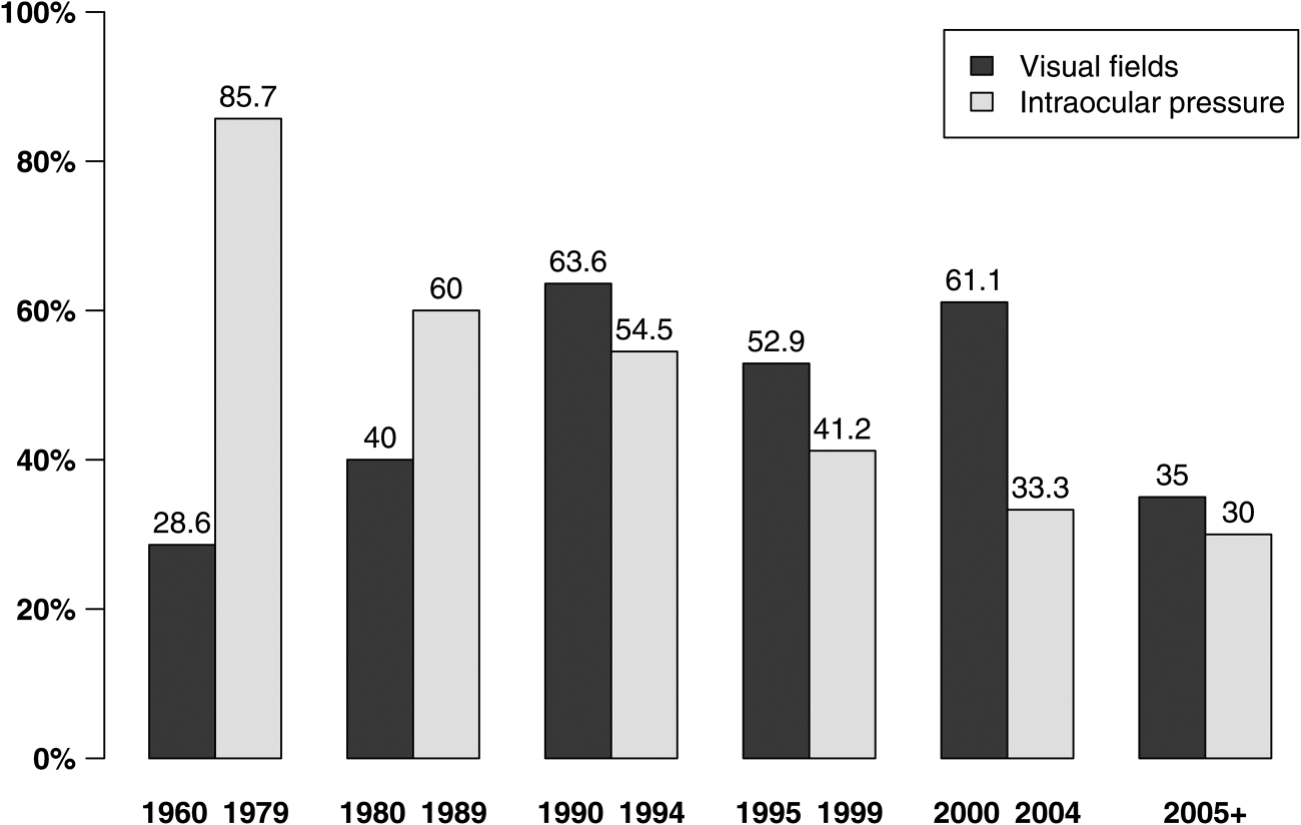
Proportion of studies over time performing visual fields on all participants or intraocular pressure to diagnose primary open angle glaucoma (POAG).

In total, 81 articles met the inclusion criteria. They examined POAG prevalence in population based surveys published between 1966 to 2014, involving 5266 cases of POAG among 216 214 individuals. Online supplementary table S1 provides the key features of each individual article included in the review, including the number of POAG cases, number of participants, age range of participants, survey years, habitation type and methods used to diagnose POAG cases. Table 1 summarises the studies contributing to the overall and gender-specific analyses, including the number of studies, POAG cases and participants by ethnic group. Most studies were carried out in White populations (36%, 29/81), with fewer studies in South Asian and East Asian (19.8%, 16/81; 18.5%, 15/81 respectively), Black (16%, 13/81), and a smaller number of studies in South-East Asian (6%, 5/81), Hispanic or Latino (2.5%, 2/81) and mixed ethnic populations (table 1). Table 2 examines the effect of important population and study level covariates on the risk of POAG, including age, ethnicity, year of survey and methods of POAG diagnosis. The risk of POAG increased with age across all ethnic groups. As in our previous review,^5^ we modelled ethnic-specific associations with age. The risk of POAG per decade in age was highest among Hispanics (2.3 times greater risk per decade in adjusted analyses), followed by White populations with a doubling of risk, South Asians (1.7 greater risk per decade), Black populations and South-East Asians (1.6 greater risk per decade), and lowest among East Asians (with approximately a 1.5 times greater risk per decade) (table 2). Men showed 33% higher risk of POAG (OR=1.33, 95% CrI 1.24, 1.42), with no evidence that the effect of gender differed across ethnic groups. This effect remained after adjustment for other study covariates, including age and study design factors. Older studies had progressively lower risk of POAG, and historic studies (1960–1979) report significantly lower POAG prevalence than studies after year 2000 (OR=0.45, 95% CrI 0.24, 0.89). However, given shifts in diagnostic methods over time (figure 1), adjustment for year of survey and study methods (including the extent of VF testing and/or use of an IOP criterion), was not carried out due to concerns over collinearity. In sensitivity analysis, there was no evidence of an effect of calendar year on POAG risk after year 2000. In terms of study methods, there was no evidence that ISGEO diagnosis modified POAG risk among studies published over the period when guidelines were introduced (ie, post 2002).^6^ However, in all studies, including those published before ISGEO guidelines, reliance on IOP in POAG diagnosis reduced the POAG prevalence compared with studies routinely performing VFs on all participants to diagnose POAG (table 2). While this effect was attenuated with adjustment for other study covariates, the effect was marginally stronger when studies including IOP assessment in diagnosis were pooled (40 study populations, unadjusted pooled OR 0.59, 95% CrI 0.41, 0.88; adjusted pooled OR 0.78, 95% CrI 0.58, 1.01). Figure 1 shows the proportion of studies performing VFs on all participants or relying on IOP to obtain POAG diagnosis over time. A shift away from relying on IOP to diagnose POAG is clearly shown. While studies have increasingly undertaken VF testing on all participants to diagnose POAG over time, fewer studies used routine VF testing on all participants from 2005 onwards.

**Table 1:** Summary of the number of study populations with data on primary open angle glaucoma (POAG) prevalence by ethnic group

Ethnicity	Study	K	N	n	Survey years	
	Populations				Range	Mean*
Prevalence reported in men and women combined						
White	29	107	60 465	1188	1963–2002	1987
Black	13	68	24 258	1363	1966–2009	1995
East Asian	15	38	56 400	1182	1985–2010	2002
South Asian	16	50	44 384	743	1990–2008	2002
South-East Asian	5	10	9302	254	1997–2005	2002
Hispanic or Latino	2	11	10 916	385	1998–2003	2001
Other/mixed	8	17	10 489	151	1969–2011	2002
Prevalence reported in men and women separately (subset of all studies)						
White	20	148	40 012	706	1963–2002	1989
Black	6	36	15 249	1028	1986–2009	1997
East Asian	12	66	45 711	690	1988–2010	2002
South Asian	8	58	25 308	480	1998–2008	2003
South-East Asian	3	12	6604	187	1997–2005	2001
Hispanic or Latino	2	12	10 929	385	1998–2003	2001
Other/Mixed	3	18	3069	21	1969–1992	1985

K: Total number of available estimates of prevalence.

N: Total number of participants (published or estimated).

n: Total number of cases of POAG (published or estimated).

*Mean survey year weighted by study population size.

**Table 2:** ORs of primary open angle glaucoma (POAG) for a decade increase in age, trends over time and study design factors

			All surveys	Surveys conducted since 2000
Factor	Study	Unadjusted OR*	Adjusted OR†	Adjusted OR†
	populations	(95% CrI)	(95% CrI)	(95% CrI)
Effect per decade increase in age by racial group				
White	29	1.99 (1.86, 2.13)	1.99 (1.86, 2.12)	1.97 (1.50, 2.64)
Black	13	1.60 (1.52, 1.67)	1.59 (1.52, 1.67)	1.47 (1.38, 1.57)
East Asian	15	1.48 (1.39, 1.57)	1.48 (1.39, 1.57)	1.45 (1.34, 1.57)
South Asian	16	1.69 (1.58, 1.82)	1.69 (1.58, 1.81)	1.70 (1.57, 1.83)
South-East Asian	5	1.56 (1.31, 1.87)	1.56 (1.31, 1.88)	1.46 (1.22, 1.75)
Hispanic or Latino	2	2.31 (2.12, 2.52)	2.31 (2.12, 2.52)	2.24 (2.03, 2.48)
Other/mixed	8	1.88 (1.44, 2.47)	1.90 (1.45, 2.52)	1.42 (0.85, 2.32)
Year of survey				
1960–1979	7	0.45 (0.24, 0.89)		
1980–1989	15	0.75 (0.46, 1.22)		
1990–1999	28	0.81 (0.54, 1.22)		
2000+	38	1.00		
Study design factors: visual field (VF)/intraocular pressure (IOP)				
VF on all	37	1.00	1.00	
VF on all and IOP criterion	5	0.55 (0.26, 1.18)	0.64 (0.35, 1.14)	
IOP criterion and VF on subset	35	0.58 (0.40, 0.86)	0.78 (0.56, 0.99)	
Other	11	0.90 (0.52, 1.53)	1.23 (0.77, 1.76)	
*Study design factors*				
(In surveys conducted since 2000)				
Follows ISGEO and VF on all	10	1.00		1.00
Follows ISGEO and VF on subset	14	0.70 (0.37, 1.34)		0.69 (0.44, 1.10)
Does not follow ISGEO	14	1.03 (0.53, 1.91)		0.79 (0.48, 1.33)
Sex‡				
Female	54	1.00	1.00	
Male	54	1.33 (1.24, 1.42)	1.30 (1.22, 1.41)	

Study design factors: VF/IOP.

VF on all—Identification of POAG included VF assessment on all participants and IOP was not used as a defining criterion of POAG.

VF on all and IOP criterion—Identification of POAG included VF assessment on all participants and IOP was used as a defining criterion of POAG.

IOP criterion and VF on subset—Identification of POAG did not include VF assessment on all participants and IOP was used as a defining criterion of POAG. In this group, 30/32 studies performed VF on a subset of participants only, the remaining 2 studies^21^ ^22^ did not undertake VF testing.

Other—Identification of POAG did not include VF assessment on all participants nor did POAG case definition rely on IOP criteria. In this group nine studies performed VF on a subset of participants only; one study^23^ did not perform VF testing at all.

Study design factors: ISGEO.

Follow ISGEO and VF on all—Study design follows the ISGEO criteria and VF assessment was performed on all participants.

Follow ISGEO and VF on subset—Study design follows the ISGEO criteria and VF assessment was performed on a subset of participants (high-risk or proportion/consecutive).

Does not follow ISGEO—A more conventional method of determining glaucoma using a combination of optic disc features and VF defects.

*ORs are not mutually adjusted but take into account the clustering of prevalence estimates within study populations.

†ORs are mutually adjusted for all factors listed in this column, and allowing for the clustering of prevalence estimates within study populations.

‡Analysis performed on a subset of data that report prevalence by sex.

CrI, credible intervals; ISGEO, International Society for Geographical and Epidemiological Ophthalmology.

**Figure 2:**
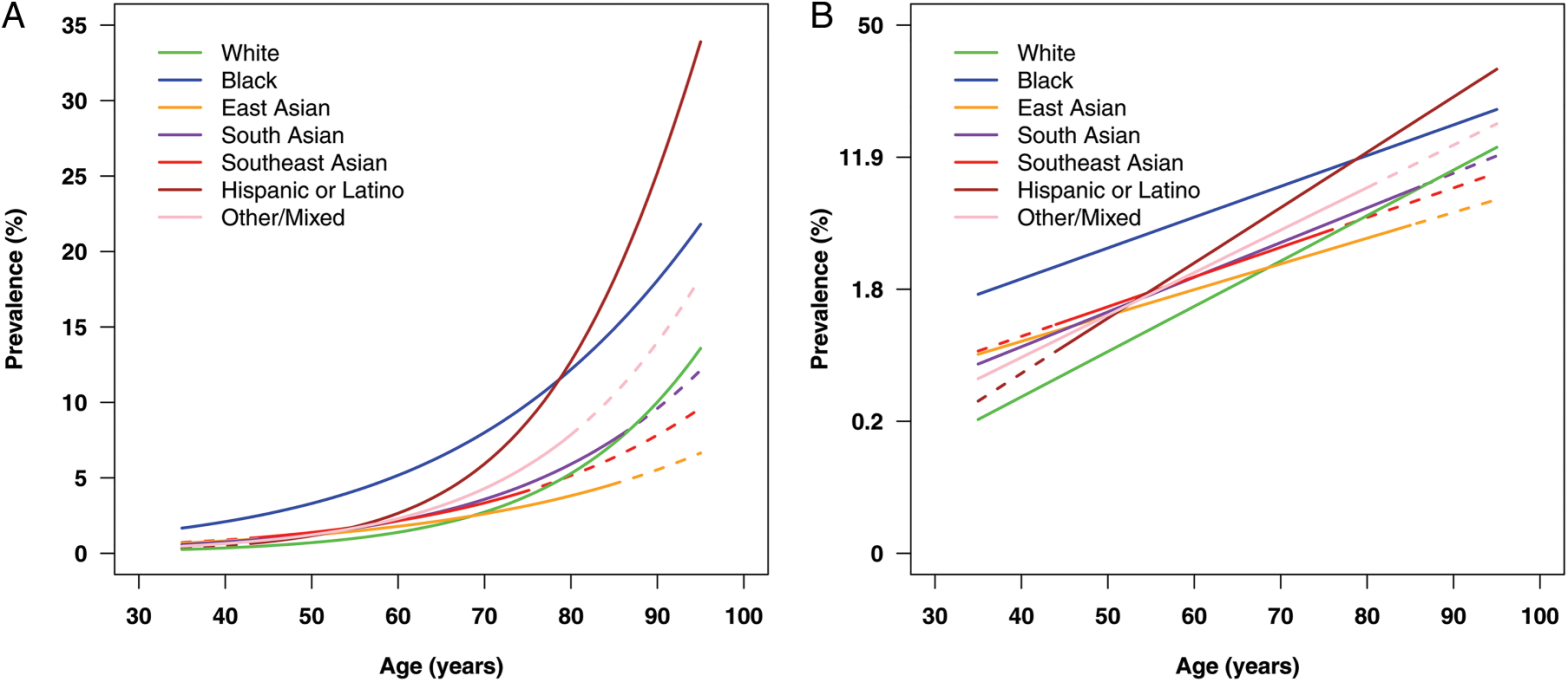
Estimated prevalence (%) of primary open angle glaucoma with age for men and women combined by ethnicity; (A) shows prevalence on the y axis on the normal scale, (B) on the log scale. Coloured lines come from regression models adjusting for age (log-linear relationship), fitted separately for White (green), Black (blue), East Asian (orange), South Asian (purple), Southeast Asian (navy), Hispanic or Latino (brown), and other or mixed ethnicity (pink) groups. Solid lines are given across the age range of available data for each ethnic group.

Ethnic specific prevalence of POAG by age is given in table 3. Estimates are standardised to surveys that performed VF but not IOP assessment on all participants. The corresponding estimates for White men and White women, along with prevalence by year of age with greater granularity are given in online supplementary table S2. All ethnic groups showed a log-linear increase in POAG prevalence with age but the slope of the log-linear association differed by ethnic group (figure 2). While the age-dependent increase in POAG prevalence is highest for White populations and Hispanics, Black populations have the highest absolute levels of POAG prevalence at each age, except above age 80 years, where the prevalence is highest among Hispanics (table 3, figure 2).

**Table 3:** Estimated prevalence of primary open angle glaucoma (POAG) by age and ethnicity in men and women combined

	Prevalence of POAG by age and ethnicity (%)						
Age (years)	White	Black	East Asian	South Asian	South-East Asian	Hispanic or Latino	Other/Mixed
35	0.3 (0.2, 0.4)	1.7 (1.2, 2.4)	0.7 (0.4, 1.0)	0.6 (0.4, 0.9)	0.7 (0.3, 1.5)*	0.3 (0.1, 0.8)*	0.5 (0.2, 1.0)
40	0.4 (0.2, 0.5)	2.1 (1.5, 3.0)	0.8 (0.5, 1.2)	0.8 (0.5, 1.1)	0.9 (0.4, 1.8)*	0.5 (0.2, 1.2)*	0.6 (0.3, 1.3)
45	0.5 (0.4, 0.7)	2.6 (1.9, 3.8)	1.0 (0.7, 1.5)	1.0 (0.7, 1.4)	1.1 (0.6, 2.2)	0.8 (0.3, 1.7)	0.9 (0.5, 1.6)
50	0.7 (0.5, 1.0)	3.3 (2.4, 4.7)	1.2 (0.8, 1.8)	1.3 (0.9, 1.8)	1.4 (0.8, 2.6)	1.2 (0.5, 2.6)	1.2 (0.7, 2.1)
55	1.0 (0.7, 1.4)	4.1 (3.0, 5.8)	1.5 (1.0, 2.1)	1.7 (1.2, 2.3)	1.7 (1.0, 3.1)	1.8 (0.8, 3.8)	1.7 (1.0, 2.8)
60	1.4 (1.0, 1.9)	5.2 (3.7, 7.2)	1.8 (1.2, 2.6)	2.1 (1.5, 3.0)	2.2 (1.2, 3.9)	2.7 (1.2, 5.7)	2.3 (1.4, 3.8)
65	2.0 (1.5, 2.7)	6.4 (4.7, 9.0)	2.2 (1.5, 3.1)	2.8 (2.0, 3.9)	2.7 (1.5, 4.8)	4.0 (1.8, 8.4)	3.2 (1.8, 5.3)
70	2.7 (2.1, 3.7)	8.0 (5.8, 11.1)	2.6 (1.8, 3.8)	3.6 (2.5, 5.0)	3.3 (1.9, 6.0)	5.9 (2.7, 12.2)	4.3 (2.4, 7.7)
75	3.8 (2.9, 5.1)	9.9 (7.2, 13.6)	3.2 (2.1, 4.6)	4.6 (3.2, 6.5)	4.2 (2.3, 7.5)	8.7 (4.0, 17.4)	5.8 (3.0, 11.1)
80	5.3 (4.0, 7.1)	12.2 (8.9, 16.6)	3.8 (2.6, 5.6)	5.9 (4.1, 8.4)	5.1 (2.8, 9.5)*	12.7 (6.0, 24.4)	7.9 (3.7, 16.0)
85	7.3 (5.5, 9.8)	14.9 (10.9, 20.2)	4.6 (3.1, 6.8)	7.6 (5.2, 10.8)	6.4 (3.3, 12.1)*	18.2 (8.7, 33.1)	10.5 (4.5, 22.7)*
90	10.0 (7.4, 13.5)	18.1 (13.4, 24.3)	5.5 (3.7, 8.2)*	9.6 (6.6, 13.8)*	7.8 (3.9, 15.3)*	25.2 (12.7, 43.1)	14.0 (5.4, 31.3)*
95	13.6 (10.1, 18.1)	21.8 (16.2, 29.0)	6.6 (4.4, 9.9)*	12.1 (8.2, 17.5)*	9.6 (4.5, 19.3)*	33.9 (18.0, 53.7)	18.3 (6.6, 41.6)*

Estimates correspond to prevalence (%) and 95% credible intervals,and are standardised to surveys that performed visual field but not intraocular pressure assessment on all participants.

*Estimates obtained by extrapolation from the fitted model.

Although we observed weak evidence of a higher odds of POAG in populations from urban compared with rural populations, once we adjusted for the ethnic-specific associations with age there was no evidence of habitation type on POAG prevalence and the OR was remarkably close to 1.0 (data not presented).

Estimates of the global POAG prevalence by region were attained by applying modelled age and ethnic-specific prevalence estimates to UN defined population data in 5-yearly intervals from age 40 years onwards for calendar years 2015, 2020 and 2025 (table 4). The number of cases and population prevalence of POAG are shown. Global estimates suggest a burden of 57.5 million (95% CI 46.4 to 73.1 million) POAG cases in 2015, rising to 65.5 million (95% CrI 52.8, 83.2 million) in 2020. While the prevalence of POAG is highest in Africa (4.5%), nearly half of POAG cases (49%) are in Asia due to its considerably larger population size. Although the age-specific prevalence is stable over time, the overall prevalence of POAG is predicted to increase by 0.1–0.2% from 2015 to 2020 because of population ageing. The global share of POAG cases will increase in Latin America, Asia and Africa but decrease in Europe and North America, due to more rapid expansion of the ageing population in these countries of non-European ancestry. Maps showing global estimates of the number of POAG cases over time (for 2015, 2020 and 2025) are provided as online supplementary figures S2–S4. Online supplementary figures S2–S4 show changes in prevalence over time.

**Table 4:** Global primary open angle glaucoma (POAG) trends: age, gender and ethnic-specific prevalence estimates applied to United Nations (UN) defined population data for age above 40 years for 2010, 2015 and 2020

UN population	Total population (≥40 years)			POAG cases (95% credible intervals)			Population prevalence (%)			Percentage of global prevalence			Change from 2015 to 2020		Change from 2015 to 2025	
	2015	2020	2025	2015	2020	2025	2015	2020	2025	2015	2020	2025	Population (%)	Global (%)	Population (%)	Global (%)
Europe	385.84	396.76	407.88	7.81 (5.92, 10.52)	8.30 (6.30, 11.18)	8.82 (6.69, 11.88)	2.0	2.1	2.2	13.6	12.7	11.8	0.1	−0.9	0.2	−1.8
Africa	225.86	264.24	311.83	10.13 (7.34, 14.14)	11.83 (8.57, 16.51)	13.93 (10.09, 19.44)	4.5	4.5	4.5	17.6	18.1	18.7	0	0.5	0	1.1
Asia	1558.06	1725.14	1907.99	28.53 (21.64, 37.54)	32.51 (24.70, 42.73)	36.98 (28.13, 48.56)	1.8	1.9	1.9	49.6	49.7	49.6	0.1	0.1	0.1	0
Western Asia	68.86	82.72	98.57	0.95 (0.71, 1.28)	1.14 (0.86, 1.54)	1.37 (1.04, 1.86)	1.4	1.4	1.4	1.6	1.7	1.8	0	0.1	0	0.2
Central Asia	18.62	21.02	23.90	0.30 (0.20, 0.43)	0.34 (0.23, 0.49)	0.39 (0.26, 0.56)	1.6	1.6	1.6	0.5	0.5	0.5	0	0	0	0
Eastern Asia	753.46	802.14	853.84	13.06 (8.88, 18.90)	14.51 (9.88, 21.00)	16.05 (10.93, 23.24)	1.7	1.8	1.9	22.7	22.2	21.5	0.1	−0.5	0.2	−1.2
Southern Asia	511.15	584.30	665.59	9.92 (7.06, 13.91)	11.50 (8.19, 16.13)	13.33 (9.49, 18.69)	1.9	2.0	2.0	17.2	17.6	17.9	0.1	0.4	0.1	0.7
South-Eastern Asia	205.97	234.95	266.09	4.03 (2.31, 7.17)	4.72 (2.70, 8.37)	5.49 (3.14, 9.74)	2.0	2.0	2.1	7.0	7.2	7.4	0	0.2	0.1	0.4
Northern America	171.08	180.56	190.70	3.30 (2.50, 4.45)	3.67 (2.79, 4.95)	4.10 (3.11, 5.52)	1.9	2.0	2.1	5.7	5.6	5.5	0.1	−0.1	0.2	−0.2
Latin America and the Caribbean	209.76	238.23	268.01	7.07 (3.69, 13.40)	8.34 (4.33, 15.84)	9.85 (5.09, 18.69)	3.4	3.5	3.7	12.3	12.7	13.2	0.1	0.4	0.3	0.9
Central America	51.28	59.56	67.97	1.59 (0.73, 3.18)	1.92 (0.89, 3.83)	2.32 (1.07, 4.62)	3.1	3.2	3.4	2.8	2.9	3.1	0.1	0.1	0.3	0.3
Southern America	142.57	161.53	181.53	4.65 (2.15, 9.27)	5.50 (2.54, 10.94)	6.49 (3.01, 12.89)	3.3	3.4	3.6	8.1	8.4	8.7	0.1	0.3	0.3	0.6
Caribbean	15.90	17.14	18.51	0.83 (0.60, 1.14)	0.92 (0.67, 1.28)	1.03 (0.75, 1.42)	5.2	5.4	5.6	1.4	1.4	1.4	0.2	0	0.4	0
Oceania	15.87	17.28	18.86	0.31 (0.24, 0.41)	0.36 (0.27, 0.47)	0.41 (0.31, 0.54)	2.0	2.1	2.2	0.5	0.5	0.5	0.1	0	0.2	0
Australia and New Zealand	13.29	14.29	15.45	0.26 (0.20, 0.35)	0.29 (0.22, 0.39)	0.33 (0.25, 0.45)	1.9	2.1	2.2	0.5	0.4	0.4	0.2	−0.1	0.3	−0.1
Melanesia	2.20	2.57	2.96	0.04 (0.03, 0.07)	0.05 (0.03, 0.08)	0.06 (0.04, 0.10)	1.9	2.0	2.0	0.1	0.1	0.1	0.1	0	0.1	0
Micronesia	0.17	0.19	0.20	0.004 (0.002, 0.006)	0.004 (0.003, 0.007)	0.005 (0.003, 0.009)	2.1	2.3	2.6	0.0	0.0	0.0	0.2	0	0.5	0
Polynesia	0.22	0.24	0.26	0.005 (0.003, 0.008)	0.006 (0.003, 0.010)	0.007 (0.004, 0.011)	2.3	2.4	2.6	0.0	0.0	0.0	0.1	0	0.3	0
Global	2566.47	2822.22	3105.27	57.54 (46.44, 73.07)	65.46 (52.84, 83.17)	74.62 (60.20, 94.85)	2.2	2.3	2.4	100.0	100.0	100.0	0.1	0	0.2	0

Total population and numbers of POAG cases are reported in millions.

In Europe and North America the predominant ethnicity was assumed to be White.

In Africa the predominant ethnicity was assumed to be Black.

Asia includes Western Asia (White), Central Asia (East Asian), Eastern Asia (East Asian), Southern Asia (South Asian) and South-Eastern Asia (South-East Asian).

Latin America and the Caribbean include the Caribbean (Black), Central America (Hispanic) and South America (Hispanic).

Oceania includes Australia and New Zealand (White), Melanesia (Other/Mixed), Micronesia (Other/Mixed) and Polynesia (Other/Mixed).

## Discussion

This systematic review and meta-analysis represents the most up-to-date estimates of POAG prevalence and is based on the most comprehensive data available. Compared with similar analyses, it includes twice as many studies, participants, and POAG cases than studies published nearly a decade ago (81 vs 34 studies^2^ and 46 studies,^5^ 216 214 vs 103 567 participants,^5^ and 5266 vs 2509 cases^5^ at best) and a half more cases and participants to the most recent meta-analysis (with 3370 cases, 140 496 participants).^3^ It also encompasses a greater ethnic diversity of study populations. Due to a recent surge in Asian population studies (23 studies reporting on 24 distinct Asian populations since 2005), the data allow the subdivision of results by South, East and South-Eastern Asia, and more accurate prevalence estimates in this region due to the larger numbers. This is particularly important as the worldwide POAG case burden is greatest in Asia.

The review affirms that Black populations have the highest POAG prevalence from early middle life.^4^ ^5^ This suggests that exposure to disease is longer in Black populations, and may explain the observation that glaucoma is more severe in Black populations compared with White populations in an age-matched comparisons of elderly patients.^11^ Age-specific increase in POAG prevalence is highest among White populations and Hispanic populations (Hispanics appear to overtake POAG prevalence in Black populations in later life), followed by Asians, and is lowest in Black populations. Reasons for these apparent ethnic differences are unclear, and may relate to differences in anatomy, pigmentation and/or genetic susceptibility.^12^ They may also relate to ethnic differences in susceptibility to other non-communicable diseases and their precursors.^13–15^ For example, Black populations and Asians are at greater risk compared with White populations of cardiometabolic disease (eg, stroke, diabetes, coronary heart disease and their associated precursors), and these are putative risk factors for POAG.^16^ ^17^ It is noteworthy that as with POAG, the magnitude of these ethnic differences are also highly dependent on age.^13^

Accurate modelling of POAG by age and by ethnic group is important when obtaining global estimates, as any error is magnified when applied to global population numbers. This is particularly relevant at older ages where log-linear increases in POAG compete with increasing mortality. Equally, it is important to standardise for age when comparing POAG prevalence between populations, to ensure that any apparent population differences are not confounded by age. While numbers with POAG are predicted to increase due to population ageing, application of the same POAG prevalence rates to 2015, 2020 and 2025 demographic data assume that the underlying age-specific prevalence remains stable, and that any potential changes in diagnostic technology and definitions will not alter the underlying rate of detection. While this assumption can be assumed for the next decade (table 4 and online supplementary figures S2–S4), we believe that further reviews are needed to extrapolate findings beyond 2025.

Our results show that POAG prevalence decreases with older studies, and that year of survey is closely linked to changing trends in study designs and diagnostic definitions. Historic studies (1960–1979) often include raised IOP as a diagnostic criterion (figure 1), and they report significantly lower POAG prevalence than studies after year 2000, which may be due to missed cases of low tension glaucoma. The trend for routine VF testing on all participants also changes with time (figure 1). While VF results form part of the POAG case definitions in every study included, only those conducted in 1990–2004 tended to perform VF test on all subjects (59%, ie, 27 out of 46 studies). After 2005, that drops to only 35%. The lack of complete VF data is likely to cause underdiagnosis, as most POAG case definitions require the simultaneous presence of structural and functional changes to optic disc and VF. The move away from routine VF testing on all participants could be linked to the adoption of ISGEO guidelines, published in 2002.^6^ These guidelines aim to improve and homogenise the diagnosis of POAG by defining a ‘normal’ optic disc using the study populations’ own cup-to-disc ratio and applying statistical cut-offs at 97.5th and 99.5th centiles for glaucomatous VF changes. They allow for three diagnostic categories depending on the level of evidence available. Crucially, those without VF testing can be diagnosed with POAG under the ISGEO scheme, albeit under a category of lower level of evidence and certainty. This might have encouraged investigators to limit time-consuming and costly VF testing to high-risk patients only. However, incomplete VF data will affect the accuracy of ISGEO diagnosis, as the criteria assume that all subjects had attempted to perform VF. Those who could not satisfactorily perform VF due to poor visual function will be diagnosed on more stringent optic disc changes (CDR>99.5th centile rather than 97.5th centile), but any patients with glaucoma with normal fields who did not undergo VF testing by design will also be diagnosed on this more stringent criteria. Moreover, the definition of the ‘normal population’ in ISGEO is open to interpretation, and could refer to those without glaucomatous field changes, to ‘hypernormals’ with repeated normal VF tests. As such, the availability of VF data could bias the definitions of normal. These limitations need to be considered carefully in future population surveys. Our findings corroborate with our expectations, that studies which performed VF on all subjects and did not rely on an IOP criterion, report a higher odds of POAG. Among ISGEO studies, the completeness of VF testing is associated with higher odds of POAG.

Although earlier systematic reviews have suggested no gender difference after age adjustment,^4^ other reviews suggest that higher prevalence in women^2^ ^18^ is commensurate with clinical representation of the disease. Our finding affirms our earlier work, and findings from a recent review,^3^ in showing higher age-adjusted prevalence in men.^5^ Our findings are now based on far greater numbers with 3497 POAG cases out of 146 882 participants with gender-specific data, compared with 1355 and 61 267 in our earlier review,^5^ and with comparable numbers to those used in a more recent review.^3^ Hence, we can be increasingly confident that age-adjusted prevalence is higher in men compared with women, and that this finding remains consistent across all ethnic groups. As we indicated in our earlier work this gender difference remains even when meta-analytical data suggesting higher prevalence in women are included,^5^ and it would take an extraordinarily large study with opposite results to alter the overall findings. Biological reasons for this gender difference may reflect anatomical difference in retinal nerve fibre thickness,^19^ or potentially protective hormonal effects among women.^20^ Higher POAG prevalence in men is also akin to well known gender differences in other non-communicable diseases, such as cardiovascular disease, which is explained by greater lifestyle and biological risk factors among men; risk factors that have also been shown to be positively associated with glaucoma.^16^

In contrast to a previous recent review,^3^ our analyses do not support an association between POAG prevalence and habitation type. However, we allowed for the association with age to vary by ethnicity and it is likely that the effect of habitation type observed in the previous review was due to residual confounding.

This review has a number of strengths and limitations. The larger number of studies reflects the inclusive approach adopted, which allows all studies with potentially relevant data to contribute to the meta-analysis. In contrast to the most recent review,^3^ our estimates were standardised to studies with optimal methods, while allowing studies with suboptimal methods to contribute to pooled estimates compensating for study differences. Adopting a more exclusive approach, that is, omitting studies with imperfect study methods, would result in loss of power and would not allow the effect of study differences to be quantified. Consequent to our large data set, our estimates were associated with greater accuracy. For example, previous estimates of the global number of POAG cases suggests 44.1 million cases in 2013, but the 95% CIs range from 31.3 million to 60.9 million, representing a twofold difference.^3^ Our estimate of 57.5 million global POAG cases in 2015, is comparable to this earlier estimate (ie, the point estimate is contained within the CI),^3^ but has a narrower 95% CrI of 46.4 million to 73.1 million (ie, a 1.6-fold difference). The same occurs for 2020, where we produce a similar but more precise estimate than earlier estimates.^3^ The improved accuracy is important to the appropriate planning of health service provision, especially as earlier estimates could result in a potential doubling or halving of economic costs.^3^

Limitations of this study include the omission of study response rates in the analysis as reliable data were difficult to obtain, and that formal participation rates (ie, the number with data/number invited) were not routinely reported. However, our earlier work suggests that non-response has a minimal effect on POAG estimates,^5^ and agrees with other work^3^ that could not elicit a reliable estimate for response rates. Second, the use of ethnicity-based estimates to generate global and regional POAG prevalence means that multiethnic populations will not be adequately represented by our estimates based on the single predominant ethnic group of the region. In addition, it assumes that populations of the same ethnicity settled in different geographical locations share similar POAG risks, which conflicts with the understanding that POAG susceptibility is a product of environmental factors and genetics. Despite these caveats we believe our estimates provide greater certainty than comparable studies.^3^

In summary, this meta-analysis provides the most comprehensive and current evidence on POAG prevalence. The results corroborate with the previous predicted trend of more rapid increases in POAG cases in developing countries on non-European ancestry, due to more aged population expansion, and reiterate the finding of greater prevalence among men than women, and among Black populations and older Hispanic populations. However, the changing study design methods and glaucoma definitions through time highlight the difficulty researchers had and still experience in attempting to adequately define POAG. The study provides clear recommendations for the use and interpretation of study methods, particularly ISGEO guidelines,^6^ in the future.

## Supplementary Material

Web supplement
