# Supplementary material for: Global variations and time trends in the prevalence of primary open angle glaucoma (POAG): a systematic review and meta-analysis
Source: Br J Ophthalmol. 2015 Aug 18;100(1):86–93. doi: 10.1136/bjophthalmol-2015-307223 (PMC4717368; doi:10.1136/bjophthalmol-2015-307223)
Supplement: Web supplement [file bjophthalmol-2015-307223-s1.pdf]

Title: Global variations and time trends in the prevalence of Primary Open Angle Glaucoma (POAG); a systematic review and meta-analysis

Authors: Venediktos V Kapetanakis,[1*] Michelle P Y Chan,[2*] Paul J Foster,[2,3] Derek G Cook,[1] Christopher G Owen,[1] Alicja R Rudnicka[1]

1. Population Health Research Institute, St George's, University of London, Cranmer Terrace, London, SW17 0RE, UK

2. Division of Genetics and Epidemiology, UCL Institute of Ophthalmology, 11-43 Bath Street, London EC1V 9EL, UK

3. NIHR Biomedical Research Centre Moorfields Eye Hospital NHS Foundation Trust, 162 City Road, London EC1V 2PD, UK.

* Joint first authors

Address for correspondence:- Dr Alicja R Rudnicka, as above, tel: +44 (0)20 87252799, fax: +44 (0)20 87253584, e-mail: arudnick@sgul.ac.uk

Supplemental Material

STATISTICAL APPENDIX: BAYSIAN MODELS FOR THE ESTIMATION OF POAG PREVALENCE

Prevalence of POAG for both genders combined, adjusted for multiple risk factors

Some surveys report POAG prevalence or number of POAG cases for the whole population sample whilst other studies report results in finer granularity (e.g. by age group, ethnicity, habitation type and sex). All regression models were based on data stratified by ethnicity with the finest possible granularity with respect to age and habitation type. When data were reported separately by age and habitation type but not by both age and habitation type, preference was given to stratification by age.

Let I be the total number of clusters defined by the combination of published article, survey year and geographical location as explained in the statistical analysis Section. For each $i \in \{1, \dots, I\}$, let K_i be the total number of estimates of POAG prevalence in cluster i. For each $i \in \{1, \dots, I\}$ and $k_i \in \{1, \dots, K_i\}$, let n_{ik_i} denote the number of POAG cases within population i of sample size N_{ik_i}, and $p_{ik_i} = \frac{n_{ik_i}}{N_{ik_i}}$ denote the corresponding prevalence estimate. Our main regression model is described by the following formulas:

$$n_{ik_i} \sim Binomial(p_{ik_i}, N_{ik_i})$$

$$\text{logit}(p_{ik_i}) = \beta_0 + \beta_A A_{k_i} + \sum_{m=1}^{6} \beta_{E^{(m)}} E_{k_i}^{(m)} + \sum_{m=1}^{6} \beta_{AE^{(m)}} A_{k_i} E_{k_i}^{(m)} + \sum_{m=1}^{3} \beta_{D^{(m)}} D_{k_i}^{(m)} + U_i$$

$$U_i \sim N(0, \sigma^2)$$

for $k_i \in \{1, \dots, K_i\}$ and $i \in \{1, \dots, I\}$, where A is age; $E^{(1)}, E^{(2)}, E^{(3)}, E^{(4)}, E^{(5)}, E^{(6)}$ are indicator variables for East Asian, South Asian, South-East Asian, black, Hispanic or Latino, and mixed or other ethnic populations, respectively; $D^{(1)}, D^{(2)}, D^{(3)}$ are indicator variables for surveys in which identification of POAG included visual field assessment on all participants and intra ocular pressure was used as a defining criterion of POAG, surveys in which identification of POAG did not include visual field assessment on all participants and intra ocular pressure was used as a defining criterion of POAG, and surveys in which identification of POAG did not include visual field assessment on all participants nor did POAG case definition rely on intra ocular pressure criteria, respectively; and U_i are random effects taking into account the hierarchical structure of the data according to which estimates of prevalence are nested within clusters. The model allows a different log-linear association between age and prevalence of POAG by ethnicity. Low-information prior distributions were used for the quantity $\tau = 1/\sigma^2$ and all other regression coefficients.

Other regression models

Additional regression models investigating the independent associations between the prevalence of POAG and i) age by ethnicity, iii) survey year, iv) study design factors based on use of visual fields and intraocular pressure assessment, v) study design factors based on ISGEO classification and vi) sex were carried out following a similar approach.

POAG prevalence and 95% credible intervals by ethnicity and age

Median POAG prevalence estimates and associated 95% credible intervals were obtained from Model 6, standardising to studies that routinely used VFs on all participants to diagnose POAG. For each ethnic group m, the distribution of POAG prevalence at age A was obtained by:

$$p(A, m) = 1/(1 + \exp(-\beta_0 - \beta_A A - \beta_{E^{(m)}} E^{(m)} - \beta_{AE^{(m)}} AE^{(m)} - \epsilon))$$

$$\epsilon \sim N\left(0, \frac{\sigma^2}{I}\right)$$

where A is age and $E^{(1)}, E^{(2)}, E^{(3)}, E^{(4)}, E^{(5)}, E^{(6)}$ are indicator variables for East Asian, South Asian, South-East Asian, black, Hispanic or Latino, and mixed or other ethnic populations, respectively.

PROGRAMMING CODE

The OpenBUGS and R programming code for the implementation of all models is available from the authors upon request.

SUPPLEMENTAL TABLES

Table legends

Supplemental Table S1. Articles contributing to the meta-analysis

Supplemental Table S2. Estimated prevalence of POAG by age (yearly), gender and ethnicity

Supplemental Table S1. Articles contributing to the meta-analysis

Ethnicity	Author	Study name / Location, Country	Publication Year	Participant Numbers	POAG cases	Gender			Age range	Habitation type	ISGEO	VF/IOP
						M	F	B				
White	Hollows [24]	Ferndale, Wales, UK	1966	4231	20	•	•	•	40-74	Rural	3	2
	Bankes [25]	Bedford, UK	1968	5941	45			•	20+	Urban	3	2
	Leibowitz [26]	Framingham, USA	1980	2631	50	•	•	•	52-85	Mixed	3	2
	Bengtsson [27]	Dalby, Sweden	1981	1511	13			•	58-69	Rural	3	0
	Martinez [28]	Gisborne, New Zealand	1982	481	20	•	•	•	65+	Urban	3	2
	Gibson [29]	Melton Mowbray, UK	1985	484	32	•	•	•	76+	Urban	3	2
	Ringvold [30]	Middle-Norway	1991	1871	63			•	65+	Urban	3	2
	Tielsch [12]	Baltimore, MD, USA	1991	2913	32			•	40+	Urban	3	0
	Klein [31]	Beaver Dam, WI, USA	1992	4926	104	•	•	•	43+	Rural	3	1
	Coffey [32]	Roscommon, Ireland	1993	2186	41	•	•	•	50+	Rural	3	2
	Dielemans [33]	Rotterdam, The Netherlands	1994	3062	34	•	•	•	55+	Urban	3	1
	Leske [34]	Barbados, West Indies	1994	133	1	•	•	•	40-86	Mixed	3	0
	Giuffre [35]	Casteldaccia, Sicily, Italy	1995	1062	13	•	•	•	40-99	Rural	3	2
	Hirvela [23]	Oulu, Finland	1995	500	27			•	70-95	Rural	3	3
	Ekstrom [36]	Tierp, Sweden	1996	760	29	•	•	•	65-74	Urban	3	0
	Mitchell [37]	Blue Mountains, Australia	1996	3655	87			•	49+	Urban	3	0
	Cedrone [38]	Ponza, Italy	1997	1034	26	•	•	•	40+	Rural	3	2
	Bonomi [39]	Egna-Neumarkt, Italy	1998	4297	84	•	•	•	40+	Rural	3	0
	Reidy [40]	North London, UK	1998	1547	47	•	•	•	65-100	Urban	3	0
	Kozobolis [41]	Crete, Greece	2000	1107	31	•	•	•	40+	Mixed	3	2
	Weih[42]	Melbourne VIP, Australia	2001	3264	56	•	•	•	40+	Urban	3	1
	Weih[42]	Melbourne VIP, Australia	2001	1469	29	•	•	•	40+	Rural	3	1
	Anton [43]	Segovia, Spain	2004	510	10	•	•	•	40-79	Urban	3	0
	Nizankowska [44]	Wroclaw, Poland	2005	4853	62	•	•	•	40-79	Urban	3	2
	Friedman [11]	Salisbury, MD, USA	2006	929	79			•	73+	Urban	3	0
	Astrom [45]	Skelleftea, Sweden	2007	339	1	•	•	•	66	Urban	3	3

Ethnicity	Author	Study name / Location, Country	Publication Year	Participant Numbers	POAG cases	Gender			Age range	Habitation type	ISGEO	VF/IOP
						M	F	B				
	Sakata [46]	Piraquara City, Brazil	2007	1170	25	•	•	•	40+	Urban	2	2
	Topouzis [47]	Thessaloniki Eye Study, Greece	2007	2554	98			•	60+	Urban	3	0
	Arnarsson [48]	Reykjavik, Iceland	2009	1045	29			•	50+	Urban	2	3
Black	Wallace [49]	Jamaica, West Indies	1969	574	6			•	35-74	Mixed	3	1
	Mason [50]	St. Lucia, West Indies	1989	1679	147	•	•	•	30+	Mixed	3	2
	Tielsch [12]	Baltimore, MD, USA	1991	2395	100			•	40+	Urban	3	0
	Leske [34]	Barbados, West Indies	1994	4498	308	•	•	•	40-86	Mixed	3	0
	Wormald [51]	London, UK	1994	873	32	•	•	•	35+	Urban	3	0
	Buhrmann [52]	Kongwa, East Africa	2000	3247	99			•	40+	Rural	3	0
	Ekwerekwu [53]	Alum-Inyi, Nigeria	2002	664	14			•	30+	Rural	3	2
	Rotchford [54]	Kwazulu-Natal, South Africa	2002	1005	28			•	40+	Rural	1	0
	Rotchford [55]	Temba, South Africa	2003	839	31			•	40-97	Urban	1	0
	Ntim-Amponsah [56]	Ghana, West Africa	2004	1785	149	•	•	•	30-100	Mixed	3	2
	Friedman [11]	Salisbury, MD, USA	2006	285	57			•	73+	Urban	3	0
	Ashaye [57]	Southwestern Nigeria	2013	811	50	•	•	•	40+	Urban	2	3
	Budenz [58]	Tema Eye Survey	2013	5603	342	•	•	•	40+	Urban	1	0
East Asian	Hu [21]	Shunyi, Beijing, China	1989	3000	1			•	40+	Urban	3	2
	Shiose [59]	Japan	1991	8924	224	•	•	•	30+	Rural	3	2
	Foster [60]	Hovsgol, Mongolia	1996	942	5	•	•	•	40-89	Rural	3	0
	Iwase [61]	Tajimi, Japan	2004	3021	119	•	•	•	40+	Urban	3	0
	He [62]	Liwan District, Guangzhou, China	2006	1405	29	•	•	•	50-93	Urban	2	2
	Ohkubo [63]	Komatsu City, Japan	2007	1128	58			•	40+	Urban	2	3
	Wang [64]	Beijng Eye Study, China	2010	4315	111	•	•	•	40+	Mixed	1	0
	Kim CS [65]	Namil-myon, South Korea	2011	1532	55	•	•	•	40+	Rural	1	0
	Kim JH [66]	Sangju, South Korea	2011	671	18	•	•	•	50+	Urban	2	2
	Liang [67]	Handan, China	2011	6716	125	•	•	•	30+	Rural	2	3
	Song [68]	Kailu County, Tongliao, Mongolia	2011	5158	73	•	•	•	40+	Rural	1	0
	Yoon[69]	Korean NHANES, South Korea	2011	6561	256			•	40+	Mixed	3	3

Ethnicity	Author	Study name / Location, Country	Publication Year	Participant Numbers	POAG cases	Gender			Age range	Habitation type	ISGEO	VF/IOP
						M	F	B				
	Sun [70]	Bin County	2012	4956	35	•	•	•	40+	Rural	2	2
	Zhong [71]	Yunnan Minority Eye Study	2012	2133	22	•	•	•	50+	Rural	2	2
	Li[72]	Yongchuan Glaucoma Study, China	2014	5938	51	•	•	•	50+	Urban	3	2
South Asian	Rauf [73]	Southall, London, UK	1994	184	5			•	30+	Urban	3	0
	Jacob [74]	Vellore, India	1998	972	4			•	30-60	Urban	3	2
	Dandona [75]	Andhra Pradesh, India	2000	1399	27			•	30-102	Urban	3	2
	Ramakrishnan [76]	Aravind, India	2003	5150	64	•	•	•	40-90	Rural	3	0
	Rahman [77]	Dhaka, Bangladesh	2004	2347	29			•	35-85	Urban	2	2
	Raychaudhuri [78]	South Parganas, India	2005	1269	38	•	•	•	50+	Rural	1	0
	Vijaya [79]	Tamil Nadu, India	2005	3924	64	•	•	•	40+	Rural	1	0
	Palimkar [80]	Chhattisgarh, India	2008	7438	37			•	35+	Rural	3	2
	Vijaya [81]	Chennai, India	2008	3850	135	•	•	•	40+	Urban	1	0
	Garudadri [82]	Andhra Pradesh, India	2010	934	37	•	•	•	40+	Urban	2	2
	Garudadri [82]	Andhra Pradesh, India	2010	2790	45	•	•	•	40+	Rural	2	2
	Sia [83]	Kandy, Sri Lanka	2010	1244	30			•	40+	Rural	2	3
	Thapa [84]	Bhaktapur Glaucoma Study	2012	3991	51	•	•	•	40+	Rural	1	0
	Nangia [85]	Central India Eye & Medical Study	2013	4570	122			•	30+	Rural	3	0
	Narayanaswamy [86]	Singaporian Indian Eye Study	2013	3400	46	•	•	•	40-80	Urban	2	2
	Rauf [87]	British Asian Community Eye Study	2013	922	9			•	40+	Urban	3	0
Southeast Asian	Foster [88]	Tanjong Pagar, Singapore	2000	1232	22	•	•	•	40-81	Urban	3	0
	Metheetrairut [89]	Bangkok, Thailand	2002	2092	73	•	•	•	60-104	Urban	3	2
	Bourne [90]	Rom Klao, Bangkok, Thailand	2003	701	16			•	50+	Urban	1	0
	Casson [91]	Meiktila, Myanmar, Burma	2007	1997	39			•	40+	Rural	2	3
	Shen [92]	Malay, Singapore	2008	3280	104	•	•	•	40-80	Urban	2	3
Hispanic or Latino	Quigley [93]	Nogales and Tucson, AZ, USA	2001	4774	94	•	•	•	41+	Urban	1	0
	Varma [94]	Los Angeles, CA, USA	2004	6142	291	•	•	•	40+	Urban	3	0
Other/Mixed	Alsbirk [22]	Eskimos, Greenland	1973	396	5	•	•	•	40+	Rural	3	2
	Arkell [95]	Eskimos, Alaska	1987	1686	1	•	•	•	15+	Rural	3	2

Ethnicity	Author	Study name / Location, Country	Publication Year	Participant Numbers	POAG cases	Gender			Age range	Habitation type	ISGEO	VF/IOP
						M	F	B				
	Salmon [96]	Mamre, South Africa	1993	987	15	•	•	•	40+	Rural	3	2
	Sakata [46]	Piraquara City, Brazil	2007	397	15			•	40+	Urban	2	2
	Al-Mansouri [97]	Qatar	2011	3149	44			•	40+	Mixed	3	2
	Landers [98]	Alice Springs, Australia	2011	1884	7			•	20+	Rural	2	3
	Pakravan [99]	Yazd Eye Study, Iran	2013	1770	51			•	40-80	Urban	1	0
	Pakravan [99]	Yazd Eye Study, Iran	2013	220	13			•	40-80	Rural	1	0

Gender

M: POAG prevalence reported for males.
F: POAG prevalence reported for females.
B: POAG prevalence reported for both sexes combined.

More information about ISGEO classification

1: “Follows ISGEO & VF on all” (Study design follows the ISGEO criteria and visual field assessment was performed on all participants).
2: “Follows ISGEO & VF on subset” (Study design follows the ISGEO criteria and visual field assessment was performed on a subset of participants limited to a proportion of subjects or only high risk subjects).
3: "Does not follow ISGEO" (A more conventional method of determining glaucoma using a combination of optic disc features & visual field defects.

More information about VF/IOP classification

0: “VF on all” (Identification of POAG included visual field assessment on all participants and intra ocular pressure was not used as a defining criterion of POAG).
1: “VF on all & IOP criterion” (Identification of POAG included visual field assessment on all participants and intra ocular pressure was used as a defining criterion of POAG).
2: “IOP criterion & VF on subset” (Identification of POAG did not include visual field assessment on all participants and intra ocular pressure was used as a defining criterion of POAG).
3: “Other” (Identification of POAG did not include visual field assessment on all participants nor did POAG case definition rely on intra ocular pressure criteria).

Supplemental Table S2. Estimated prevalence of POAG by age (yearly), gender and ethnicity

Age (years)	Prevalence of POAG by age and ethnicity (%)								
	White			Black	East Asian	South Asian	Southeast Asian	Hispanic or Latino	Other/Mixed
	Men	Women	All	All	All	All	All	All	All
35	0.3 (0.1 , 0.8)*	0.3 (0.1 , 0.7)*	0.3 (0.2 , 0.4)	1.7 (1.2 , 2.4)	0.7 (0.4 , 1.0)	0.6 (0.4 , 0.9)	0.7 (0.3 , 1.5)*	0.3 (0.1 , 0.8)*	0.5 (0.2 , 1.0)
36	0.3 (0.1 , 0.8)*	0.3 (0.1 , 0.7)*	0.3 (0.2 , 0.4)	1.7 (1.2 , 2.5)	0.7 (0.5 , 1.1)	0.6 (0.4 , 0.9)	0.7 (0.4 , 1.6)*	0.4 (0.2 , 0.8)*	0.5 (0.2 , 1.0)
37	0.3 (0.1 , 0.9)*	0.3 (0.1 , 0.8)*	0.3 (0.2 , 0.4)	1.8 (1.3 , 2.6)	0.7 (0.5 , 1.1)	0.7 (0.4 , 1.0)	0.8 (0.4 , 1.6)*	0.4 (0.2 , 0.9)*	0.5 (0.2 , 1.1)
38	0.4 (0.1 , 0.9)*	0.3 (0.1 , 0.8)*	0.3 (0.2 , 0.5)	1.9 (1.4 , 2.8)	0.8 (0.5 , 1.1)	0.7 (0.5 , 1.0)	0.8 (0.4 , 1.7)*	0.4 (0.2 , 1.0)*	0.6 (0.3 , 1.1)
39	0.4 (0.1 , 1.0)*	0.3 (0.1 , 0.9)*	0.3 (0.2 , 0.5)	2.0 (1.4 , 2.9)	0.8 (0.5 , 1.2)	0.7 (0.5 , 1.1)	0.9 (0.4 , 1.8)*	0.5 (0.2 , 1.1)*	0.6 (0.3 , 1.2)
40	0.4 (0.2 , 1.0)*	0.4 (0.1 , 0.9)*	0.4 (0.2 , 0.5)	2.1 (1.5 , 3.0)	0.8 (0.5 , 1.2)	0.8 (0.5 , 1.1)	0.9 (0.4 , 1.8)*	0.5 (0.2 , 1.2)*	0.6 (0.3 , 1.3)
41	0.4 (0.2 , 1.1)*	0.4 (0.1 , 1.0)*	0.4 (0.3 , 0.6)	2.2 (1.6 , 3.1)	0.9 (0.6 , 1.3)	0.8 (0.6 , 1.2)	0.9 (0.5 , 1.9)*	0.6 (0.2 , 1.3)*	0.7 (0.4 , 1.3)
42	0.5 (0.2 , 1.2)	0.4 (0.2 , 1.0)	0.4 (0.3 , 0.6)	2.3 (1.6 , 3.3)	0.9 (0.6 , 1.3)	0.8 (0.6 , 1.2)	1.0 (0.5 , 1.9)*	0.6 (0.3 , 1.4)*	0.7 (0.4 , 1.4)
43	0.5 (0.2 , 1.3)	0.4 (0.2 , 1.1)	0.4 (0.3 , 0.6)	2.4 (1.7 , 3.4)	0.9 (0.6 , 1.4)	0.9 (0.6 , 1.3)	1.0 (0.5 , 2.0)*	0.7 (0.3 , 1.5)*	0.8 (0.4 , 1.4)
44	0.5 (0.2 , 1.3)	0.5 (0.2 , 1.2)	0.5 (0.3 , 0.7)	2.5 (1.8 , 3.6)	1.0 (0.6 , 1.4)	0.9 (0.7 , 1.3)	1.1 (0.6 , 2.1)	0.7 (0.3 , 1.6)	0.8 (0.5 , 1.5)
45	0.6 (0.2 , 1.4)	0.5 (0.2 , 1.2)	0.5 (0.4 , 0.7)	2.6 (1.9 , 3.8)	1.0 (0.7 , 1.5)	1.0 (0.7 , 1.4)	1.1 (0.6 , 2.2)	0.8 (0.3 , 1.7)	0.9 (0.5 , 1.6)
46	0.6 (0.2 , 1.5)	0.5 (0.2 , 1.3)	0.5 (0.4 , 0.8)	2.8 (2.0 , 3.9)	1.0 (0.7 , 1.5)	1.0 (0.7 , 1.5)	1.2 (0.6 , 2.2)	0.8 (0.4 , 1.9)	1.0 (0.5 , 1.7)
47	0.6 (0.2 , 1.6)	0.5 (0.2 , 1.4)	0.6 (0.4 , 0.8)	2.9 (2.1 , 4.1)	1.1 (0.7 , 1.6)	1.1 (0.8 , 1.6)	1.2 (0.7 , 2.3)	0.9 (0.4 , 2.0)	1.0 (0.6 , 1.8)
48	0.7 (0.3 , 1.7)	0.6 (0.2 , 1.4)	0.6 (0.4 , 0.9)	3.0 (2.2 , 4.3)	1.1 (0.8 , 1.6)	1.2 (0.8 , 1.6)	1.3 (0.7 , 2.4)	1.0 (0.4 , 2.2)	1.1 (0.6 , 1.9)
49	0.7 (0.3 , 1.8)	0.6 (0.2 , 1.5)	0.7 (0.5 , 0.9)	3.2 (2.3 , 4.5)	1.2 (0.8 , 1.7)	1.2 (0.9 , 1.7)	1.3 (0.7 , 2.5)	1.1 (0.5 , 2.4)	1.1 (0.7 , 2.0)
50	0.8 (0.3 , 1.9)	0.6 (0.3 , 1.6)	0.7 (0.5 , 1.0)	3.3 (2.4 , 4.7)	1.2 (0.8 , 1.8)	1.3 (0.9 , 1.8)	1.4 (0.8 , 2.6)	1.2 (0.5 , 2.6)	1.2 (0.7 , 2.1)
51	0.8 (0.3 , 2.0)	0.7 (0.3 , 1.7)	0.8 (0.6 , 1.1)	3.5 (2.5 , 4.9)	1.3 (0.9 , 1.8)	1.3 (1.0 , 1.9)	1.4 (0.8 , 2.7)	1.3 (0.6 , 2.8)	1.3 (0.8 , 2.2)
52	0.9 (0.3 , 2.2)	0.7 (0.3 , 1.8)	0.8 (0.6 , 1.1)	3.6 (2.6 , 5.1)	1.3 (0.9 , 1.9)	1.4 (1.0 , 2.0)	1.5 (0.8 , 2.8)	1.4 (0.6 , 3.0)	1.4 (0.8 , 2.3)
53	0.9 (0.4 , 2.3)	0.8 (0.3 , 1.9)	0.9 (0.6 , 1.2)	3.8 (2.7 , 5.3)	1.4 (0.9 , 2.0)	1.5 (1.1 , 2.1)	1.6 (0.9 , 2.9)	1.5 (0.7 , 3.3)	1.5 (0.9 , 2.4)
54	1.0 (0.4 , 2.5)	0.8 (0.3 , 2.0)	0.9 (0.7 , 1.3)	4.0 (2.9 , 5.6)	1.4 (1.0 , 2.1)	1.6 (1.1 , 2.2)	1.7 (0.9 , 3.0)	1.6 (0.7 , 3.5)	1.6 (1.0 , 2.6)
55	1.1 (0.4 , 2.6)	0.9 (0.3 , 2.1)	1.0 (0.7 , 1.4)	4.1 (3.0 , 5.8)	1.5 (1.0 , 2.1)	1.7 (1.2 , 2.3)	1.7 (1.0 , 3.1)	1.8 (0.8 , 3.8)	1.7 (1.0 , 2.8)
56	1.1 (0.5 , 2.8)	0.9 (0.4 , 2.3)	1.1 (0.8 , 1.5)	4.3 (3.1 , 6.1)	1.5 (1.0 , 2.2)	1.7 (1.2 , 2.5)	1.8 (1.0 , 3.3)	1.9 (0.8 , 4.2)	1.8 (1.1 , 2.9)
57	1.2 (0.5 , 3.0)	1.0 (0.4 , 2.4)	1.1 (0.8 , 1.6)	4.5 (3.3 , 6.4)	1.6 (1.1 , 2.3)	1.8 (1.3 , 2.6)	1.9 (1.1 , 3.4)	2.1 (0.9 , 4.5)	1.9 (1.2 , 3.1)
58	1.3 (0.5 , 3.2)	1.0 (0.4 , 2.5)	1.2 (0.9 , 1.7)	4.7 (3.4 , 6.6)	1.7 (1.1 , 2.4)	1.9 (1.4 , 2.7)	2.0 (1.1 , 3.5)	2.3 (1.0 , 4.9)	2.0 (1.2 , 3.3)
59	1.4 (0.6 , 3.4)	1.1 (0.4 , 2.7)	1.3 (1.0 , 1.8)	4.9 (3.6 , 6.9)	1.7 (1.2 , 2.5)	2.0 (1.5 , 2.9)	2.1 (1.2 , 3.7)	2.4 (1.1 , 5.3)	2.2 (1.3 , 3.5)

Age (years)	Prevalence of POAG by age and ethnicity (%)								
		White		Black	East Asian	South Asian	Southeast Asian	Hispanic or Latino	Other/Mixed
	Men	Women	All	All	All	All	All	All	All
60	1.5 (0.6 , 3.6)	1.2 (0.5 , 2.8)	1.4 (1.0 , 1.9)	5.2 (3.7 , 7.2)	1.8 (1.2 , 2.6)	2.1 (1.5 , 3.0)	2.2 (1.2 , 3.9)	2.7 (1.2 , 5.7)	2.3 (1.4 , 3.8)
61	1.6 (0.6 , 3.8)	1.2 (0.5 , 3.0)	1.5 (1.1 , 2.0)	5.4 (3.9 , 7.6)	1.9 (1.3 , 2.7)	2.3 (1.6 , 3.2)	2.3 (1.3 , 4.0)	2.9 (1.3 , 6.1)	2.5 (1.5 , 4.0)
62	1.7 (0.7 , 4.0)	1.3 (0.5 , 3.2)	1.6 (1.2 , 2.2)	5.6 (4.1 , 7.9)	1.9 (1.3 , 2.8)	2.4 (1.7 , 3.3)	2.4 (1.3 , 4.2)	3.1 (1.4 , 6.6)	2.6 (1.6 , 4.3)
63	1.8 (0.7 , 4.3)	1.4 (0.6 , 3.4)	1.7 (1.3 , 2.3)	5.9 (4.3 , 8.2)	2.0 (1.4 , 2.9)	2.5 (1.8 , 3.5)	2.5 (1.4 , 4.4)	3.4 (1.5 , 7.2)	2.8 (1.7 , 4.6)
64	1.9 (0.8 , 4.6)	1.5 (0.6 , 3.6)	1.8 (1.4 , 2.5)	6.2 (4.5 , 8.6)	2.1 (1.4 , 3.0)	2.6 (1.9 , 3.7)	2.6 (1.5 , 4.6)	3.7 (1.6 , 7.7)	3.0 (1.7 , 5.0)
65	2.0 (0.8 , 4.9)	1.6 (0.6 , 3.8)	2.0 (1.5 , 2.7)	6.4 (4.7 , 9.0)	2.2 (1.5 , 3.1)	2.8 (2.0 , 3.9)	2.7 (1.5 , 4.8)	4.0 (1.8 , 8.4)	3.2 (1.8 , 5.3)
66	2.1 (0.9 , 5.2)	1.7 (0.7 , 4.0)	2.1 (1.6 , 2.8)	6.7 (4.9 , 9.4)	2.2 (1.5 , 3.3)	2.9 (2.1 , 4.1)	2.8 (1.6 , 5.0)	4.3 (1.9 , 9.0)	3.4 (1.9 , 5.7)
67	2.3 (0.9 , 5.5)	1.8 (0.7 , 4.2)	2.2 (1.7 , 3.0)	7.0 (5.1 , 9.8)	2.3 (1.6 , 3.4)	3.1 (2.2 , 4.3)	2.9 (1.7 , 5.2)	4.7 (2.1 , 9.7)	3.6 (2.0 , 6.1)
68	2.4 (1.0 , 5.8)	1.9 (0.8 , 4.5)	2.4 (1.8 , 3.2)	7.3 (5.3 , 10.2)	2.4 (1.6 , 3.5)	3.2 (2.3 , 4.6)	3.1 (1.7 , 5.4)	5.1 (2.3 , 10.5)	3.8 (2.1 , 6.6)
69	2.6 (1.1 , 6.2)	2.0 (0.8 , 4.7)	2.6 (1.9 , 3.5)	7.7 (5.6 , 10.6)	2.5 (1.7 , 3.7)	3.4 (2.4 , 4.8)	3.2 (1.8 , 5.7)	5.5 (2.5 , 11.3)	4.0 (2.2 , 7.1)
70	2.8 (1.1 , 6.6)	2.1 (0.8 , 5.0)	2.7 (2.1 , 3.7)	8.0 (5.8 , 11.1)	2.6 (1.8 , 3.8)	3.6 (2.5 , 5.0)	3.3 (1.9 , 6.0)	5.9 (2.7 , 12.2)	4.3 (2.4 , 7.7)
71	2.9 (1.2 , 7.0)	2.2 (0.9 , 5.3)	2.9 (2.2 , 4.0)	8.4 (6.1 , 11.5)	2.7 (1.8 , 3.9)	3.8 (2.7 , 5.3)	3.5 (2.0 , 6.2)	6.4 (2.9 , 13.1)	4.6 (2.5 , 8.3)
72	3.1 (1.3 , 7.4)	2.3 (1.0 , 5.6)	3.1 (2.4 , 4.2)	8.7 (6.3 , 12.0)	2.8 (1.9 , 4.1)	4.0 (2.8 , 5.6)	3.6 (2.1 , 6.5)	6.9 (3.1 , 14.1)	4.9 (2.6 , 8.9)
73	3.3 (1.4 , 7.9)	2.5 (1.0 , 5.9)	3.3 (2.5 , 4.5)	9.1 (6.6 , 12.6)	2.9 (2.0 , 4.3)	4.2 (2.9 , 5.9)	3.8 (2.1 , 6.8)	7.5 (3.4 , 15.2)	5.2 (2.7 , 9.6)
74	3.5 (1.5 , 8.4)	2.6 (1.1 , 6.2)	3.6 (2.7 , 4.8)	9.5 (6.9 , 13.1)	3.0 (2.1 , 4.4)	4.4 (3.1 , 6.2)	4.0 (2.2 , 7.1)	8.1 (3.7 , 16.3)	5.5 (2.8 , 10.3)
75	3.8 (1.6 , 8.9)	2.8 (1.1 , 6.6)	3.8 (2.9 , 5.1)	9.9 (7.2 , 13.6)	3.2 (2.1 , 4.6)	4.6 (3.2 , 6.5)	4.2 (2.3 , 7.5)	8.7 (4.0 , 17.4)	5.8 (3.0 , 11.1)
76	4.0 (1.7 , 9.4)	3.0 (1.2 , 7.0)	4.1 (3.1 , 5.5)	10.3 (7.5 , 14.2)	3.3 (2.2 , 4.8)	4.8 (3.4 , 6.9)	4.3 (2.4 , 7.9)*	9.4 (4.4 , 18.7)	6.2 (3.1 , 12.0)
77	4.3 (1.8 , 10.0)	3.1 (1.3 , 7.4)	4.3 (3.3 , 5.9)	10.8 (7.9 , 14.8)	3.4 (2.3 , 5.0)	5.1 (3.6 , 7.2)	4.5 (2.5 , 8.2)*	10.2 (4.7 , 20.0)	6.6 (3.2 , 12.9)
78	4.5 (1.9 , 10.6)	3.3 (1.3 , 7.8)	4.6 (3.5 , 6.3)	11.2 (8.2 , 15.4)	3.5 (2.4 , 5.2)	5.4 (3.7 , 7.6)	4.7 (2.6 , 8.6)*	11.0 (5.1 , 21.4)	7.0 (3.4 , 13.9)
79	4.8 (2.0 , 11.3)	3.5 (1.4 , 8.3)	5.0 (3.7 , 6.7)	11.7 (8.6 , 16.0)	3.7 (2.5 , 5.4)	5.6 (3.9 , 8.0)	4.9 (2.7 , 9.0)*	11.8 (5.5 , 22.9)	7.4 (3.5 , 14.9)
80	5.1 (2.1 , 12.0)	3.7 (1.5 , 8.7)	5.3 (4.0 , 7.1)	12.2 (8.9 , 16.6)	3.8 (2.6 , 5.6)	5.9 (4.1 , 8.4)	5.1 (2.8 , 9.5)*	12.7 (6.0 , 24.4)	7.9 (3.7 , 16.0)
81	5.5 (2.3 , 12.7)	3.9 (1.6 , 9.2)	5.6 (4.2 , 7.6)	12.7 (9.3 , 17.3)	4.0 (2.7 , 5.8)	6.2 (4.3 , 8.8)	5.4 (2.9 , 9.9)*	13.7 (6.4 , 26.0)	8.3 (3.8 , 17.2)*
82	5.8 (2.4 , 13.4)	4.2 (1.7 , 9.8)	6.0 (4.5 , 8.1)	13.2 (9.7 , 18.0)	4.1 (2.8 , 6.0)	6.5 (4.5 , 9.3)	5.6 (3.0 , 10.4)*	14.7 (7.0 , 27.7)	8.9 (4.0 , 18.4)*
83	6.2 (2.6 , 14.2)	4.4 (1.8 , 10.3)	6.4 (4.8 , 8.7)	13.8 (10.1 , 18.7)	4.3 (2.9 , 6.3)	6.8 (4.8 , 9.8)	5.8 (3.1 , 11.0)*	15.8 (7.5 , 29.4)	9.4 (4.1 , 19.8)*
84	6.6 (2.7 , 15.1)	4.7 (1.9 , 10.9)	6.9 (5.1 , 9.2)	14.3 (10.5 , 19.4)	4.4 (3.0 , 6.5)	7.2 (5.0 , 10.3)	6.1 (3.2 , 11.5)*	16.9 (8.1 , 31.2)	9.9 (4.3 , 21.2)*
85	7.0 (2.9 , 15.9)	4.9 (2.0 , 11.5)	7.3 (5.5 , 9.8)	14.9 (10.9 , 20.2)	4.6 (3.1 , 6.8)	7.6 (5.2 , 10.8)	6.4 (3.3 , 12.1)*	18.2 (8.7 , 33.1)	10.5 (4.5 , 22.7)*
86	7.4 (3.1 , 16.9)	5.2 (2.1 , 12.1)	7.8 (5.8 , 10.5)	15.5 (11.4 , 21.0)	4.8 (3.2 , 7.0)*	7.9 (5.5 , 11.3)	6.6 (3.4 , 12.6)*	19.4 (9.4 , 35.0)	11.2 (4.7 , 24.3)*

Age (years)	Prevalence of POAG by age and ethnicity (%)								
	White			Black	East Asian	South Asian	Southeast Asian	Hispanic or Latino	Other/Mixed
	Men	Women	All	All	All	All	All	All	All
87	7.9 (3.3 , 17.8)	5.5 (2.3 , 12.8)	8.3 (6.2 , 11.2)	16.1 (11.9 , 21.7)	5.0 (3.3 , 7.3)*	8.3 (5.7 , 11.9)*	6.9 (3.5 , 13.3)*	20.8 (10.2 , 36.9)	11.8 (4.8 , 25.9)*
88	8.3 (3.5 , 18.8)	5.8 (2.4 , 13.5)	8.8 (6.6 , 11.9)	16.7 (12.3 , 22.6)	5.1 (3.4 , 7.6)*	8.7 (6.0 , 12.5)*	7.2 (3.6 , 13.9)*	22.2 (10.9 , 39.0)	12.5 (5.0 , 27.7)*
89	8.8 (3.7 , 19.9)	6.2 (2.5 , 14.2)	9.4 (7.0 , 12.7)	17.4 (12.8 , 23.4)	5.3 (3.5 , 7.9)*	9.2 (6.3 , 13.1)*	7.5 (3.7 , 14.6)*	23.7 (11.8 , 41.0)	13.2 (5.2 , 29.4)*
90	9.4 (3.9 , 21.0)	6.5 (2.7 , 15.0)	10.0 (7.4 , 13.5)	18.1 (13.4 , 24.3)	5.5 (3.7 , 8.2)*	9.6 (6.6 , 13.8)*	7.8 (3.9 , 15.3)*	25.2 (12.7 , 43.1)	14.0 (5.4 , 31.3)*
91	10.0 (4.2 , 22.1)	6.9 (2.8 , 15.8)	10.7 (7.9 , 14.3)	18.8 (13.9 , 25.2)	5.7 (3.8 , 8.5)*	10.1 (6.9 , 14.5)*	8.2 (4.0 , 16.0)*	26.8 (13.7 , 45.2)	14.8 (5.6 , 33.3)*
92	10.6 (4.4 , 23.3)	7.3 (3.0 , 16.6)	11.3 (8.4 , 15.2)	19.5 (14.4 , 26.1)	6.0 (3.9 , 8.8)*	10.6 (7.2 , 15.2)*	8.5 (4.1 , 16.8)*	28.5 (14.6 , 47.3)	15.6 (5.9 , 35.3)*
93	11.2 (4.7 , 24.5)	7.7 (3.1 , 17.5)	12.0 (8.9 , 16.1)	20.3 (15.0 , 27.0)	6.2 (4.1 , 9.2)*	11.1 (7.5 , 15.9)*	8.9 (4.2 , 17.6)*	30.3 (15.7 , 49.5)	16.5 (6.1 , 37.4)*
94	11.9 (5.0 , 25.8)	8.1 (3.3 , 18.4)	12.8 (9.5 , 17.1)	21.0 (15.6 , 28.0)	6.4 (4.2 , 9.5)*	11.6 (7.8 , 16.7)*	9.2 (4.4 , 18.4)*	32.1 (16.8 , 51.6)	17.4 (6.3 , 39.5)*
95	12.6 (5.3 , 27.1)	8.6 (3.5 , 19.4)	13.6 (10.1 , 18.1)	21.8 (16.2 , 29.0)	6.6 (4.4 , 9.9)*	12.1 (8.2 , 17.5)*	9.6 (4.5 , 19.3)*	33.9 (18.0 , 53.7)	18.3 (6.6 , 41.6)*
96	13.3 (5.6 , 28.5)*	9.0 (3.7 , 20.3)*	14.4 (10.7 , 19.2)*	22.6 (16.8 , 30.0)	6.9 (4.5 , 10.3)*	12.7 (8.6 , 18.3)*	10.0 (4.7 , 20.2)*	35.8 (19.2 , 55.9)*	19.3 (6.8 , 43.8)*
97	14.1 (5.9 , 29.9)*	9.5 (3.9 , 21.4)*	15.3 (11.3 , 20.3)*	23.4 (17.5 , 31.0)	7.2 (4.7 , 10.7)*	13.3 (8.9 , 19.2)*	10.4 (4.8 , 21.1)*	37.7 (20.5 , 58.0)*	20.3 (7.1 , 46.0)*
98	14.9 (6.3 , 31.4)*	10.1 (4.1 , 22.4)*	16.2 (12.0 , 21.5)*	24.3 (18.1 , 32.0)	7.4 (4.8 , 11.1)*	13.9 (9.3 , 20.0)*	10.9 (4.9 , 22.1)*	39.8 (21.8 , 60.1)*	21.4 (7.3 , 48.3)*
99	15.7 (6.7 , 32.9)*	10.6 (4.4 , 23.6)*	17.1 (12.7 , 22.7)*	25.1 (18.8 , 33.1)*	7.7 (5.0 , 11.5)*	14.6 (9.8 , 21.0)*	11.3 (5.1 , 23.2)*	41.8 (23.2 , 62.1)*	22.5 (7.6 , 50.5)*
100	16.6 (7.0 , 34.5)*	11.2 (4.6 , 24.7)*	18.1 (13.4 , 24.0)*	26.0 (19.5 , 34.2)*	8.0 (5.2 , 11.9)*	15.2 (10.2 , 21.9)*	11.8 (5.2 , 24.2)*	43.8 (24.7 , 64.2)*	23.6 (7.9 , 52.8)*

Estimates correspond to prevalence (%) and 95% CrI, and are standardised to surveys that performed visual field assessment on all participants.
* Estimates obtained by extrapolation from the fitted model.

SUPPLEMENTAL FIGURES

Figure legends

Supplemental Figure S1: Article selection process (QUOROM statement)

Supplemental Figure S2: Global estimates of the number of POAG cases in 2015

Supplemental Figure S3: Global estimates of the number of POAG cases in 2020

Supplemental Figure S4: Global estimates of the number of POAG cases in 2025

Supplemental Figure S1: Article selection process (QUOROM statement)

5434 published studies, letter, abstracts and review articles examining the prevalence of POAG were identified from MEDLINE, EMBASE, Web of Science and Ovid alerts from January 2005 to August 2014

5282 studies excluded after title or abstract review due to: prevalence of POAG not examined; studies reported audits or hospital/clinic based data; self-reported glaucoma status

152 studies retrieved for full paper review

117 studies excluded due to: study reported incidence of glaucoma; reporting of angle closure glaucoma prevalence only; reporting of pseudoexfoliative glaucoma prevalence only; study based on self-reported questionnaire for glaucoma status; letter not reporting original data.

35 articles reporting on primary open angle glaucoma

46 articles from previous review published in 2006 [5]

81 articles reporting on 88 distinct study populations (216,214 subjects with 5266 cases of POAG aged 40+ years) with POAG prevalence data by age and ethnicity. A subset of 51 papers reporting on 54 population based samples also provided data on POAG prevalence in men and women separately (146,882 subjects with 3497 cases of POAG).

Supplemental Figure S2: Global estimates of the number of POAG cases in 2015

Global estimate of 57.5million POAG cases worldwide in 2015

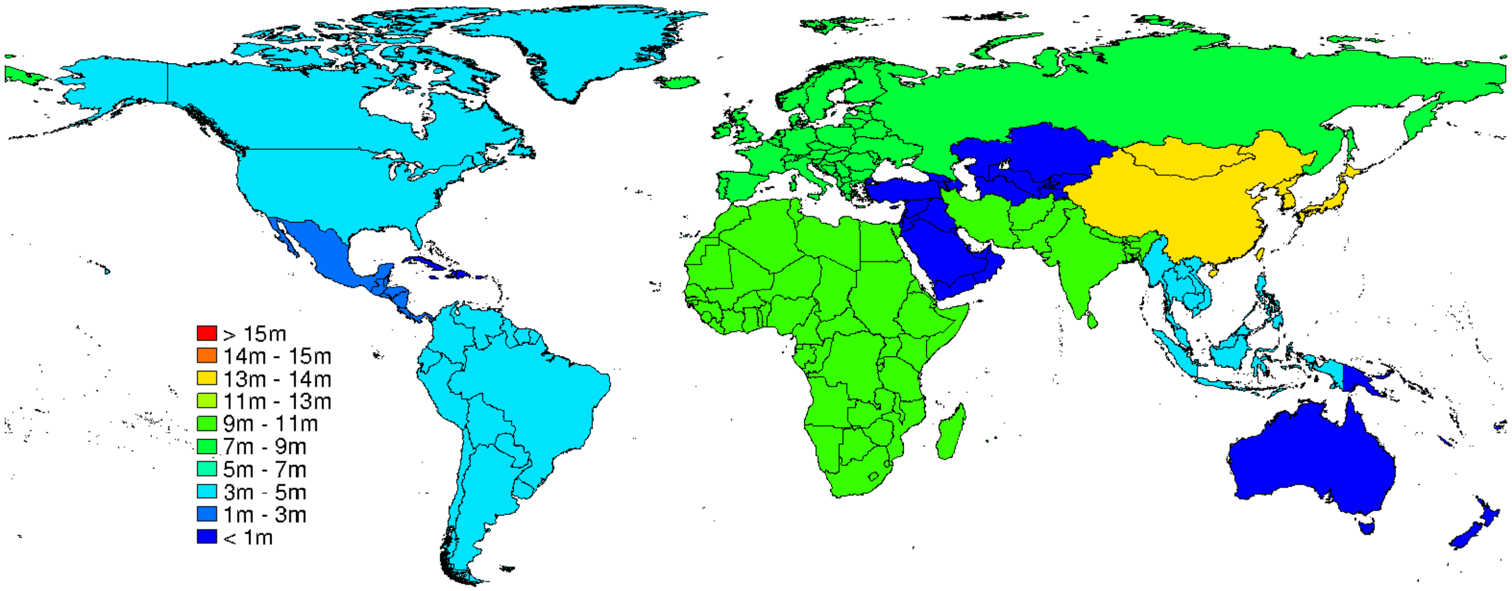

Supplemental Figure S3: Global estimates of the number of POAG cases in 2020

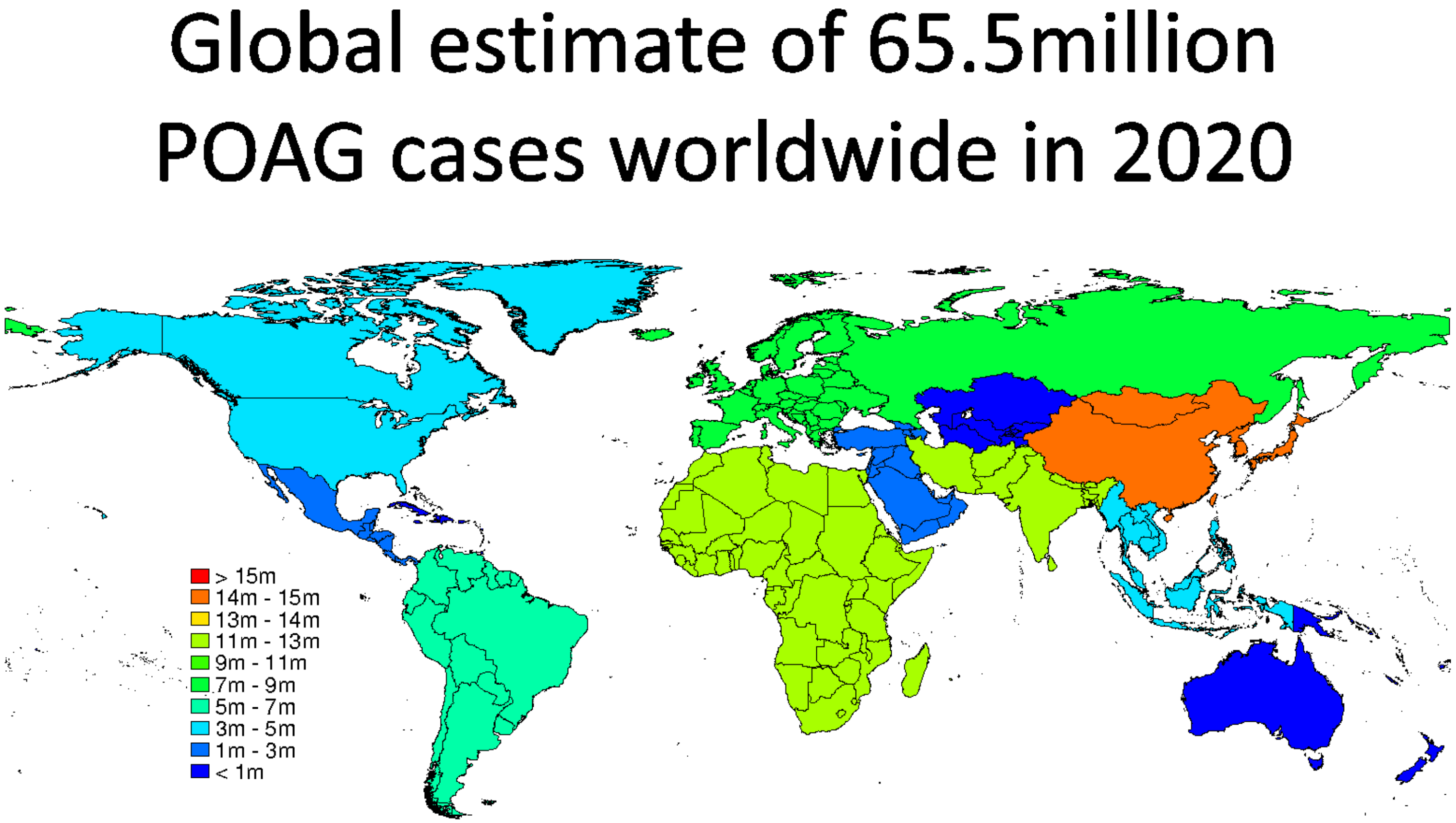

Supplemental Figure S4: Global estimates of the number of POAG cases in 2025

Global estimate of 74.6million POAG cases worldwide in 2025

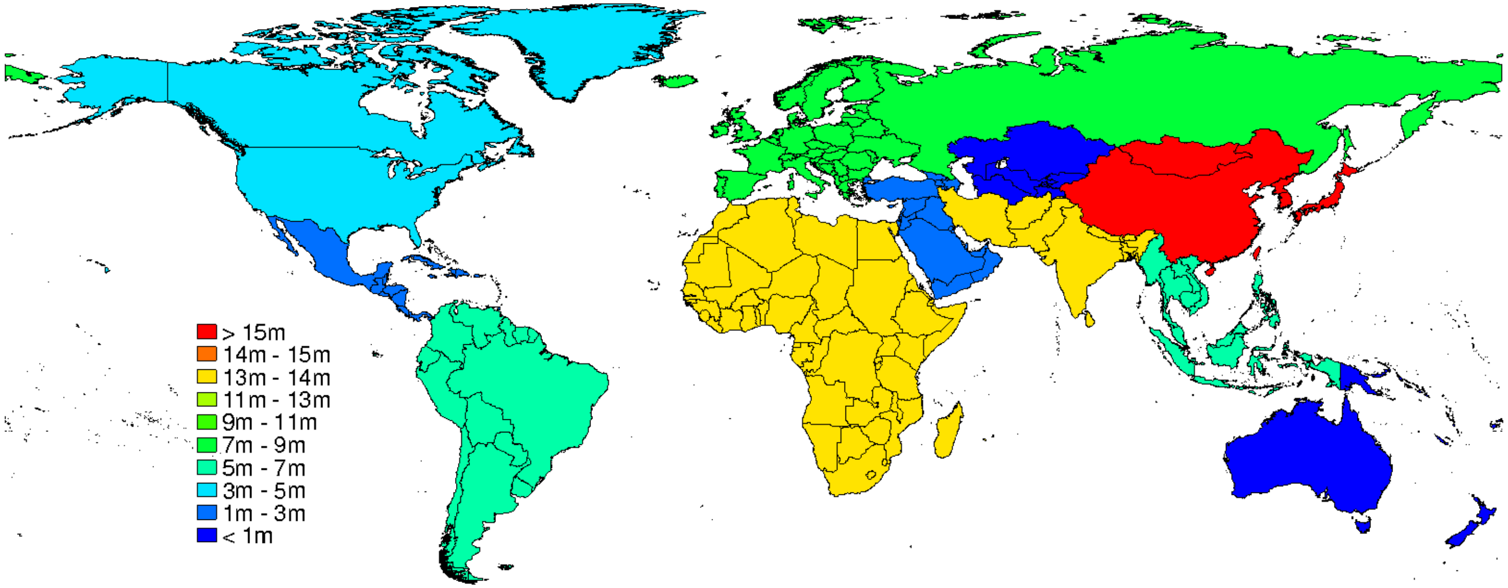

REFERENCE LIST (INCLUDING ADDITIONAL REFERENCES IN SUPPL TABLE S1)

1. World Health Organization. Visual impairment and blindness: Fact Sheet No 282. [Accessed September 2014] . 2014.
2. Quigley HA, Broman AT. The number of people with glaucoma worldwide in 2010 and 2020. *Br.J Ophthalmol.* 2006;**90**:262-7.
3. Tham YC, Li X, Wong TY, Quigley HA, Aung T, Cheng CY. Global Prevalence of Glaucoma and Projections of Glaucoma Burden through 2040: A Systematic Review and Meta-Analysis. *Ophthalmology* 2014;**121**:2081-90.
4. Friedman DS, Wolfs RC, O'Colmain BJ, Klein BE, Taylor HR, West S et al. Prevalence of open-angle glaucoma among adults in the United States. *Arch.Ophthalmol.* 2004;**122**:532-8.
5. Rudnicka AR, Mt-Isa S, Owen CG, Cook DG, Ashby D. Variations in primary open-angle glaucoma prevalence by age, gender, and race: a Bayesian meta-analysis. *Invest Ophthalmol.Vis.Sci.* 2006;**47**:4254-61.
6. Foster PJ, Buhrmann R, Quigley HA, Johnson GJ. The definition and classification of glaucoma in prevalence surveys. *Br.J Ophthalmol.* 2002;**86**:238-42.
7. Stroup DF, Berlin JA, Morton SC, Olkin I, Williamson GD, Rennie D et al. Meta-analysis of observational studies in epidemiology: a proposal for reporting. Meta-analysis Of Observational Studies in Epidemiology (MOOSE) group. *JAMA* 2000;**283**:2008-12.
8. Lunn D, Spiegelhalter D, Thomas A, Best N. The BUGS project: Evolution, critique, and future directions. *Statistics in Medicine* 2009;**28**:3049-67.

9. R Core Team. R: A Language and Environment for Statistical Computing. 2014. R Foundation for Statistical Computing, Vienna, Austria. 21-4-2015.

10. United Nations. World Population Prospects: The 2012 Revision. [Accessed September 2014] . 2012.

11. Friedman DS, Jampel HD, Munoz B, West SK. The prevalence of open-angle glaucoma among blacks and whites 73 years and older: the Salisbury Eye Evaluation Glaucoma Study. *Arch.Ophthalmol.* 2006;**124**:1625-30.

12. Tielsch JM, Sommer A, Katz J, Royall RM, Quigley HA, Javitt J. Racial variations in the prevalence of primary open-angle glaucoma. The Baltimore Eye Survey. *JAMA* 1991;**266**:369-74.

13. Chaturvedi N. Ethnic differences in cardiovascular disease. *Heart* 2003;**89**:681-6.

14. Wild SH, Fischbacher C, Brock A, Griffiths C, Bhopal R. Mortality from all causes and circulatory disease by country of birth in England and Wales 2001-2003. *J Public Health (Oxf)* 2007;**29**:191-8.

15. Roger VL, Go AS, Lloyd-Jones DM, Benjamin EJ, Berry JD, Borden WB et al. Executive summary: heart disease and stroke statistics--2012 update: a report from the American Heart Association. *Circulation* 2012;**125**:188-97.

16. Yanagi M, Kawasaki R, Wang JJ, Wong TY, Crowston J, Kiuchi Y. Vascular risk factors in glaucoma: a review. *Clin.Experiment.Ophthalmol.* 2011;**39**:252-8.

17. Lee DJ, Gomez-Marin O, Lam BL, Zheng DD. Glaucoma and survival: the National Health Interview Survey 1986-1994. *Ophthalmology* 2003;**110**:1476-83.

18. Klein R, Klein BE. The prevalence of age-related eye diseases and visual impairment in aging: current estimates. *Invest Ophthalmol.Vis.Sci.* 2013;**54**:ORSF5-ORSF13.

19. Khawaja AP, Chan MP, Garway-Heath DF, Broadway DC, Luben R, Sherwin JC et al. Associations with retinal nerve fiber layer measures in the EPIC-Norfolk Eye Study. *Invest Ophthalmol.Vis.Sci.* 2013;**54**:5028-34.

20. Pasquale LR, Rosner BA, Hankinson SE, Kang JH. Attributes of female reproductive aging and their relation to primary open-angle glaucoma: a prospective study. *J Glaucoma.* 2007;**16**:598-605.

21. Hu CN. An epidemiologic study of glaucoma in Shunyi County, Beijing. *Zhonghua Yan.Ke.Za Zhi.* 1989;**25**:115-9.

22. Alsbirk PH. Angle-closure glaucoma surveys in Greenland Eskimos. A preliminary report. *Can.J Ophthalmol.* 1973;**8**:260-4.

23. Hirvela H, Tuulonen A, Laatikainen L. Intraocular pressure and prevalence of glaucoma in elderly people in Finland: a population-based study. *Int Ophthalmol.* 1994;**18**:299-307.

24. Hollows FC, Graham PA. Intra-ocular pressure, glaucoma, and glaucoma suspects in a defined population. *Br.J Ophthalmol.* 1966;**50**:570-86.

25. Bankes JL, Perkins ES, Tsolakis S, Wright JE. Bedford glaucoma survey. *Br.Med.J* 1968;**1**:791-6.

26. Leibowitz HM, Krueger DE, Maunder LR, Milton RC, Kini MM, Kahn HA et al. The Framingham Eye Study monograph: An ophthalmological and epidemiological study

of cataract, glaucoma, diabetic retinopathy, macular degeneration, and visual acuity in a general population of 2631 adults, 1973-1975. *Surv.Ophthalmol.* 1980;**24**:335-610.

27. Bengtsson B. The prevalence of glaucoma. *Br.J Ophthalmol.* 1981;**65**:46-9.

28. Martinez GS, Campbell AJ, Reinken J, Allan BC. Prevalence of ocular disease in a population study of subjects 65 years old and older. *Am.J Ophthalmol.* 1982;**94**:181-9.

29. Gibson JM, Rosenthal AR, Lavery J. A study of the prevalence of eye disease in the elderly in an English community. *Trans.Ophthalmol.Soc.U.K.* 1985;**104 (Pt 2)**:196-203.

30. Ringvold A, Blika S, Elsas T, Guldahl J, Brevik T, Hesstvedt P et al. The middle-Norway eye-screening study. II. Prevalence of simple and capsular glaucoma. *Acta Ophthalmol.(Copenh)* 1991;**69**:273-80.

31. Klein BE, Klein R, Sponsel WE, Franke T, Cantor LB, Martone J et al. Prevalence of glaucoma. The Beaver Dam Eye Study. *Ophthalmology* 1992;**99**:1499-504.

32. Coffey M, Reidy A, Wormald R, Xian WX, Wright L, Courtney P. Prevalence of glaucoma in the west of Ireland. *Br.J Ophthalmol.* 1993;**77**:17-21.

33. Dielemans I, Vingerling JR, Wolfs RC, Hofman A, Grobbee DE, de Jong PT. The prevalence of primary open-angle glaucoma in a population-based study in The Netherlands. The Rotterdam Study. *Ophthalmology* 1994;**101**:1851-5.

34. Leske MC, Connell AM, Schachat AP, Hyman L. The Barbados Eye Study. Prevalence of open angle glaucoma. *Arch.Ophthalmol.* 1994;**112**:821-9.

35. Giuffre G, Giammanco R, Dardanoni G, Ponte F. Prevalence of glaucoma and distribution of intraocular pressure in a population. The Casteldaccia Eye Study. *Acta Ophthalmol.Scand.* 1995;**73**:222-5.

36. Ekstrom C. Prevalence of open-angle glaucoma in central Sweden. The Tierp Glaucoma Survey. *Acta Ophthalmol.Scand.* 1996;**74**:107-12.

37. Mitchell P, Smith W, Attebo K, Healey PR. Prevalence of open-angle glaucoma in Australia. The Blue Mountains Eye Study. *Ophthalmology* 1996;**103**:1661-9.

38. Cedrone C, Culasso F, Cesareo M, Zapelloni A, Cedrone P, Cerulli L. Prevalence of glaucoma in Ponza, Italy: a comparison with other studies. *Ophthalmic Epidemiol.* 1997;**4**:59-72.

39. Bonomi L, Marchini G, Marraffa M, Bernardi P, De F, I, Perfetti S et al. Prevalence of glaucoma and intraocular pressure distribution in a defined population. The Egna-Neumarkt Study. *Ophthalmology* 1998;**105**:209-15.

40. Reidy A, Minassian DC, Vafidis G, Joseph J, Farrow S, Wu J et al. Prevalence of serious eye disease and visual impairment in a north London population: population based, cross sectional study. *BMJ* 1998;**316**:1643-6.

41. Kozobolis VP, Detorakis ET, Tsilimbaris M, Siganos DS, Vlachonikolis IG, Pallikaris IG. Crete, Greece glaucoma study. *J Glaucoma.* 2000;**9**:143-9.

42. Weih LM, Nanjan M, McCarty CA, Taylor HR. Prevalence and predictors of open-angle glaucoma: results from the visual impairment project. *Ophthalmology* 2001;**108**:1966-72.

43. Anton A, Andrada MT, Mujica V, Calle MA, Portela J, Mayo A. Prevalence of primary open-angle glaucoma in a Spanish population: the Segovia study. *J Glaucoma.* 2004;**13**:371-6.

44. Nizankowska MH, Kaczmarek R. Prevalence of glaucoma in the wroclaw population. The wroclaw epidemiological study. *Ophthalmic Epidemiol.* 2005;**12**:363-71.

45. Astrom S, Linden C. Incidence and prevalence of pseudoexfoliation and open-angle glaucoma in northern Sweden: I. Baseline report. *Acta Ophthalmol.Scand.* 2007;**85**:828-31.

46. Sakata K, Sakata LM, Sakata VM, Santini C, Hopker LM, Bernardes R et al. Prevalence of glaucoma in a South brazilian population: Projeto Glaucoma. *Invest Ophthalmol.Vis.Sci.* 2007;**48**:4974-9.

47. Topouzis F, Wilson MR, Harris A, Anastasopoulos E, Yu F, Mavroudis L et al. Prevalence of open-angle glaucoma in Greece: the Thessaloniki Eye Study. *Am.J Ophthalmol.* 2007;**144**:511-9.

48. Arnarsson AM. Epidemiology of exfoliation syndrome in the Reykjavik Eye Study. *Acta Ophthalmol.* 2009;**87 Thesis 3**:1-17.

49. Wallace J, Lovell HG. Glaucoma and intraocular pressure in Jamaica. *Am.J Ophthalmol.* 1969;**67**:93-100.

50. Mason RP, Kosoko O, Wilson MR, Martone JF, Cowan CL, Jr., Gear JC et al. National survey of the prevalence and risk factors of glaucoma in St. Lucia, West Indies. Part I. Prevalence findings. *Ophthalmology* 1989;**96**:1363-8.

51. Wormald RP, Basauri E, Wright LA, Evans JR. The African Caribbean Eye Survey: risk factors for glaucoma in a sample of African Caribbean people living in London. *Eye (Lond)* 1994;**8 (Pt 3)**:315-20.

52. Buhrmann RR, Quigley HA, Barron Y, West SK, Oliva MS, Mmbaga BB. Prevalence of glaucoma in a rural East African population. *Invest Ophthalmol.Vis.Sci.* 2000;**41**:40-8.

53. Ekwerekwu CM, Umeh RE. The prevalence of glaucoma in an onchoendemic community in South-Eastern Nigeria. *West Afr.J Med.* 2002;**21**:200-3.

54. Rotchford AP, Johnson GJ. Glaucoma in Zulus: a population-based cross-sectional survey in a rural district in South Africa. *Arch.Ophthalmol.* 2002;**120**:471-8.

55. Rotchford AP, Kirwan JF, Muller MA, Johnson GJ, Roux P. Temba glaucoma study: a population-based cross-sectional survey in urban South Africa. *Ophthalmology* 2003;**110**:376-82.

56. Ntim-Amponsah CT, Amoaku WM, Ofosu-Amaah S, Ewusi RK, Idirisuriya-Khair R, Nyatepe-Coo E et al. Prevalence of glaucoma in an African population. *Eye (Lond)* 2004;**18**:491-7.

57. Ashaye A, Ashaolu O, Komolafe O, Ajayi BG, Olawoye O, Olusanya B et al. Prevalence and types of glaucoma among an indigenous African population in southwestern Nigeria. *Invest Ophthalmol.Vis.Sci.* 2013;**54**:7410-6.

58. Budenz DL, Barton K, Whiteside-de VJ, Schiffman J, Bandi J, Nolan W et al. Prevalence of glaucoma in an urban West African population: the Tema Eye Survey. *JAMA Ophthalmol.* 2013;**131**:651-8.

59. Shiose Y, Kitazawa Y, Tsukahara S, Akamatsu T, Mizokami K, Futa R et al. Epidemiology of glaucoma in Japan--a nationwide glaucoma survey. *Jpn.J Ophthalmol.* 1991;**35**:133-55.

60. Foster PJ, Baasanhu J, Alsbirk PH, Munkhbayar D, Uranchimeg D, Johnson GJ. Glaucoma in Mongolia. A population-based survey in Hovsgol province, northern Mongolia. *Arch.Ophthalmol.* 1996;**114**:1235-41.

61. Iwase A, Suzuki Y, Araie M, Yamamoto T, Abe H, Shirato S et al. The prevalence of primary open-angle glaucoma in Japanese: the Tajimi Study. *Ophthalmology* 2004;**111**:1641-8.

62. He M, Foster PJ, Ge J, Huang W, Zheng Y, Friedman DS et al. Prevalence and clinical characteristics of glaucoma in adult Chinese: a population-based study in Liwan District, Guangzhou. *Invest Ophthalmol.Vis.Sci.* 2006;**47**:2782-8.

63. Ohkubo S, Takeda H, Higashide T, Sasaki T, Sugiyama K. A pilot study to detect glaucoma with confocal scanning laser ophthalmoscopy compared with nonmydriatic stereoscopic photography in a community health screening. *J Glaucoma.* 2007;**16**:531-8.

64. Wang YX, Xu L, Yang H, Jonas JB. Prevalence of glaucoma in North China: the Beijing Eye Study. *Am.J Ophthalmol.* 2010;**150**:917-24.

65. Kim CS, Seong GJ, Lee NH, Song KC. Prevalence of primary open-angle glaucoma in central South Korea the Namil study. *Ophthalmology* 2011;**118**:1024-30.

66. Kim JH, Kang SY, Kim NR, Lee ES, Hong S, Seong GJ et al. Prevalence and characteristics of glaucoma among Korean adults. *Korean J Ophthalmol.* 2011;**25**:110-5.

67. Liang YB, Friedman DS, Zhou Q, Yang X, Sun LP, Guo LX et al. Prevalence of primary open angle glaucoma in a rural adult Chinese population: the Handan eye study. *Invest Ophthalmol.Vis.Sci.* 2011;**52**:8250-7.

68. Song W, Shan L, Cheng F, Fan P, Zhang L, Qu W et al. Prevalence of glaucoma in a rural northern china adult population: a population-based survey in kailu county, inner mongolia. *Ophthalmology* 2011;**118**:1982-8.

69. Yoon KC, Mun GH, Kim SD, Kim SH, Kim CY, Park KH et al. Prevalence of eye diseases in South Korea: data from the Korea National Health and Nutrition Examination Survey 2008-2009. *Korean J Ophthalmol* 2011;**25**:421-33.

70. Sun J, Zhou X, Kang Y, Yan L, Sun X, Sui H et al. Prevalence and risk factors for primary open-angle glaucoma in a rural northeast China population: a population-based survey in Bin County, Harbin. *Eye (Lond)* 2012;**26**:283-91.

71. Zhong H, Li J, Li C, Wei T, Cha X, Cai N et al. The prevalence of glaucoma in adult rural Chinese populations of the Bai nationality in Dali: the Yunnan Minority Eye Study. *Invest Ophthalmol.Vis.Sci.* 2012;**53**:3221-5.

72. Li H, Zhang YY, Liu SC, He XG, Li CJ, Li CH et al. Prevalence of open-angle glaucoma in southwestern China: the Yongchuan Glaucoma study. *J Huazhong.Univ Sci.Technolog.Med.Sci.* 2014;**34**:137-41.

73. Rauf A, Ong PS, Pearson RV, Wormald RP. A pilot study into the prevalence of ophthalmic disease in the Indian population of Southall. *J R.Soc.Med.* 1994;**87**:78-9.

74. Jacob A, Thomas R, Koshi SP, Braganza A, Muliyil J. Prevalence of primary glaucoma in an urban south Indian population. *Indian J Ophthalmol.* 1998;**46**:81-6.

75. Dandona L, Dandona R, Srinivas M, Mandal P, John RK, McCarty CA et al. Open-angle glaucoma in an urban population in southern India: the Andhra Pradesh eye disease study. *Ophthalmology* 2000;**107**:1702-9.

76. Ramakrishnan R, Nirmalan PK, Krishnadas R, Thulasiraj RD, Tielsch JM, Katz J et al. Glaucoma in a rural population of southern India: the Aravind comprehensive eye survey. *Ophthalmology* 2003;**110**:1484-90.

77. Rahman MM, Rahman N, Foster PJ, Haque Z, Zaman AU, Dineen B et al. The prevalence of glaucoma in Bangladesh: a population based survey in Dhaka division. *Br.J Ophthalmol.* 2004;**88**:1493-7.

78. Raychaudhuri A, Lahiri SK, Bandyopadhyay M, Foster PJ, Reeves BC, Johnson GJ. A population based survey of the prevalence and types of glaucoma in rural West Bengal: the West Bengal Glaucoma Study. *Br.J Ophthalmol.* 2005;**89**:1559-64.

79. Vijaya L, George R, Paul PG, Baskaran M, Arvind H, Raju P et al. Prevalence of open-angle glaucoma in a rural south Indian population. *Invest Ophthalmol.Vis.Sci.* 2005;**46**:4461-7.

80. Palimkar A, Khandekar R, Venkataraman V. Prevalence and distribution of glaucoma in central India (Glaucoma Survey 2001). *Indian J Ophthalmol.* 2008;**56**:57-62.

81. Vijaya L, George R, Baskaran M, Arvind H, Raju P, Ramesh SV et al. Prevalence of primary open-angle glaucoma in an urban south Indian population and comparison with a rural population. The Chennai Glaucoma Study. *Ophthalmology* 2008;**115**:648-54.

82. Garudadri C, Senthil S, Khanna RC, Sannapaneni K, Rao HB. Prevalence and risk factors for primary glaucomas in adult urban and rural populations in the Andhra Pradesh Eye Disease Study. *Ophthalmology* 2010;**117**:1352-9.

83. Sia DI, Edussuriya K, Sennanayake S, Senaratne T, Selva D, Casson RJ. Prevalence of and risk factors for primary open-angle glaucoma in central Sri Lanka: the Kandy eye study. *Ophthalmic Epidemiol.* 2010;**17**:211-6.

84. Thapa SS, Paudyal I, Khanal S, Twyana SN, Paudyal G, Gurung R et al. A population-based survey of the prevalence and types of glaucoma in Nepal: the Bhaktapur Glaucoma Study. *Ophthalmology* 2012;**119**:759-64.

85. Nangia V, Jonas JB, Matin A, Bhojwani K, Sinha A, Kulkarni M et al. Prevalence and associated factors of glaucoma in rural central India. The Central India Eye and Medical Study. *PLoS.One.* 2013;**8**:e76434.

86. Narayanaswamy A, Baskaran M, Zheng Y, Lavanya R, Wu R, Wong WL et al. The prevalence and types of glaucoma in an urban Indian population: the Singapore Indian Eye Study. *Invest Ophthalmol.Vis.Sci.* 2013;**54**:4621-7.

87. Rauf A, Malik R, Bunce C, Wormald R. The British Asian community eye study: outline of results on the prevalence of eye disease in British Asians with origins from the Indian subcontinent. *Indian J Ophthalmol.* 2013;**61**:53-8.

88. Foster PJ, Oen FT, Machin D, Ng TP, Devereux JG, Johnson GJ et al. The prevalence of glaucoma in Chinese residents of Singapore: a cross-sectional population survey of the Tanjong Pagar district. *Arch.Ophthalmol.* 2000;**118**:1105-11.

89. Metheetrairut A, Singalavanija A, Ruangvaravate N, Tuchinda R. Evaluation of screening tests and prevalence of glaucoma: integrated health research program for the Thai elderly. *J Med.Assoc.Thai.* 2002;**85**:147-53.

90. Bourne RR, Sukudom P, Foster PJ, Tantisevi V, Jitapunkul S, Lee PS et al. Prevalence of glaucoma in Thailand: a population based survey in Rom Klao District, Bangkok. *Br.J Ophthalmol.* 2003;**87**:1069-74.

91. Casson RJ, Newland HS, Muecke J, McGovern S, Abraham L, Shein WK et al. Prevalence of glaucoma in rural Myanmar: the Meiktila Eye Study. *Br.J Ophthalmol.* 2007;**91**:710-4.

92. Shen SY, Wong TY, Foster PJ, Loo JL, Rosman M, Loon SC et al. The prevalence and types of glaucoma in malay people: the Singapore Malay eye study. *Invest Ophthalmol.Vis.Sci.* 2008;**49**:3846-51.

93. Quigley HA, West SK, Rodriguez J, Munoz B, Klein R, Snyder R. The prevalence of glaucoma in a population-based study of Hispanic subjects: Proyecto VER. *Arch.Ophthalmol.* 2001;**119**:1819-26.

94. Varma R, Ying-Lai M, Francis BA, Nguyen BB, Deneen J, Wilson MR et al. Prevalence of open-angle glaucoma and ocular hypertension in Latinos: the Los Angeles Latino Eye Study. *Ophthalmology* 2004;**111**:1439-48.

95. Arkell SM, Lightman DA, Sommer A, Taylor HR, Korshin OM, Tielsch JM. The prevalence of glaucoma among Eskimos of northwest Alaska. *Arch.Ophthalmol.* 1987;**105**:482-5.

96. Salmon JF, Mermoud A, Ivey A, Swanevelder SA, Hoffman M. The prevalence of primary angle closure glaucoma and open angle glaucoma in Mamre, western Cape, South Africa. *Arch.Ophthalmol.* 1993;**111**:1263-9.

97. Al-Mansouri FA, Kanaan A, Gamra H, Khandekar R, Hashim SP, Al QO et al. Prevalence and determinants of glaucoma in citizens of qatar aged 40 years or older: a community-based survey. *Middle East Afr.J Ophthalmol* 2011;**18**:141-9.

98. Landers J, Henderson T, Craig J. The prevalence of glaucoma in indigenous Australians within Central Australia: the Central Australian Ocular Health Study. *Br.J Ophthalmol.* 2012;**96**:162-6.

99. Pakravan M, Yazdani S, Javadi MA, Amini H, Behroozi Z, Ziaei H et al. A population-based survey of the prevalence and types of glaucoma in central Iran: the Yazd eye study. *Ophthalmology* 2013;**120**:1977-84.
